# Supplementary material for: New-Generation Liquid Crystal Materials for Application in Infrared Region
Source: Materials (Basel). 2021 May 17;14(10):2616. doi: 10.3390/ma14102616 (PMC8156505; doi:10.3390/ma14102616)
Supplement: Supplementary file 1 [file materials-14-02616-s001.zip › materials-1217579-supplementary.pdf]

Supplementary Materials

A new generation liquid crystal materials for applications in infrared region

by Piotr Harmata and Jakub Herman

Synthesis experimental procedures:

4-(trifluoromethoxy)benzoic acid 2
The solution of 1-bromo-4-(trifluoromethoxy)benzene **1** (185,3g – 0.77mol) in 750cm^3 of anhydrous THF was added dropwise to magnesium chips (18.7g – 0.77mol) with vigorous stirring under an N_2 atmosphere. After addition, the reaction mixture was refluxed for 1h, then it was cooled slowly to room temperature and then to −70°C. Maintaining vigorous stirring, carbon dioxide (CO_2) was introduced with nitrogen, regulating its flow so as to maintain the temperature below -68°C. When no temperature increase was observed during the CO_2 addition, the reaction was deemed complete. Then it was stirred for 1h at -70°C and was allowed to heat up to room temperature. The solvent was then poured over the precipitate and concentrated on a rotary vacuum evaporator. The remainder was transferred to a 5000cm^3 beaker. Then 3000cm^3 of water was added and 100 cm^3 of concentrated hydrochloric acid was added with stirring. Stirred for 4 hours and filtered. The precipitate was washed with plenty of water and hexane. Yield 140,9g (89,0%). Mp = 153 – 153.5°C.

4-(trifluoromethoxy)benzoyl chloride 3
4-(trifluoromethoxy)benzoic acid **2** (80.0g; 0.390mol) in 500 ml of toluene were added to the reaction. Oxalyl chloride (35.0 cm^3; 0.408mol) and one drop of DMF were added in one portion. After mixing was started, 1 drop of DMF was added. The reaction was carried out for 24h at room temperature. Then excess oxalyl chloride with toluene (60-80ml) was distilled off and cooled to room temperature. The crude product was poured into a volumetric flask and the concentration was determined (C = 0.78mol/dm^3). The crude product was used for further reactions.

4-iodophenol 5
4-aminophenol **4** (124.1g – 1.38mol) was added to the reaction vessel. Mixing was started and the previously prepared solution of water and 96% H_2SO_4 acid (1200cm^3 H_2O and 102cm^3 96% H_2SO_4) was added dropwise. When the amine dissolved, the mixture was cooled to 0÷5°C on dry ice – acetone bath (dry ice was added in portions to maintain the set temperature range) and solution of sodium nitrite (78.6g – 1.38mol) in 170cm^3 of water was added dropwise so that the temperature did not exceed 5°C. After addition of sodium nitrite, reaction mixture was stirred for 1h. A solution of potassium iodide (207.9g – 1.25mol) in 210cm^3 of water was added dropwise so that the temperature did not exceed 5°C. After dropping the whole, reaction mixture was stirred for 0.5h. Then it was heated to a temperature of 75°C and held for 1h. After cooling to room temperature, 800cm^3 of dichloromethane was added and stirred for 10 minutes. The Na_2SO_3 solution was used to decolorize the mixture. Organic layer was washed three times with water, separated, dried with $MgSO_4$ and solvent

evaporated. Product was distilled under reduced pressure (p = 20mmHg, T= 156 – 158°C). Yield 202.9g (66.6%).

4'-(trifluoromethoxy)-[1,1'-biphenyl]-4-ol 7a

4-iodophenol **5** (25,5g – 0.116mol), 2-[4-(trifluoromethoxy)phenyl]-1,3,2-dioxaborinane **6a** (29.5g – 0.121mol), anhydrous potassium carbonate K_2CO_3 (40.0g – 0.290mol), acetone 210cm^3 and 70cm^3 water were added to reaction vessel. Reaction was carried out under an N_2 atmosphere. The mixture was stirred and heated up to the boiling temperature and kept under this conditions for 15 minutes. Then it was cooled down to 40°C and catalyst Pd(OAc)$_2$ (0.3%mol) was added in one portion. The reaction mixture was heated under reflux for 2h and sample was analysed on GC-MS to check that all iodo-derivatives had reacted. Reaction mixture was poured into 120cm^3 5% HCl solution. Crude product was extracted with dichloromethane. Organic layer was washed three times with water, separated, dried with $MgSO_4$ and solvent evaporated. The solid product was recrystallised from the hexane Yield 20.8g (70,5%). Mp = 142,0°C.

4'-(trifluoromethoxy)-[1,1'-biphenyl]-4-yl 4-(trifluoromethoxy)benzoate 8a

4'-(trifluoromethoxy)-[1,1'-biphenyl]-4-ol **7a** (2.87g; 11.0mmol), 4-(trifluoromethoxy)benzoyl chloride **3** (14.7cm^3; 11.5mmol) and 100cm^3 of toluene were added to the reaction vessel. Then pyridine (2.0cm^3; 22.0mmol) was added dropwise. An exothermic effect was observed. Stirred for 24h at room temperature. Then it was poured into a 2% solution of hydrochloric acid (50 cm^3), stirred for 1h and filtered. Moved to the separatory funnel. The organic layer was washed three times with distilled water, while the aqueous layer was washed once with toluene. The combined organic layers were dried over anhydrous $MgSO_4$. Toluene was concentrated on a rotary vacuum evaporator and the resulting solid was recrystallized from ethanol. Then the compound was purified by column chromatography on silica gel using hexane as eluent and recrystallized from ethanol. Yield: 2.6 g (52%). Mp = 144.4°C.

4-iodophenyl 4-(trifluoromethoxy)benzoate 9

4-iodophenol **5** (46.8g; 0.213mol) and 4-(trifluoromethoxy)benzoyl chloride **3** (287.0cm^3; 0.224mol) were added to the reaction vessel. Then pyridine (34.2 cm^3; 0.426 mol) was added dropwise. An exothermic effect was observed. Stirred for 24h at room temperature. Then it was poured into a 2% solution of hydrochloric acid (500 cm^3), stirred for 1h and filtered. Moved to the separatory funnel. The organic layer was washed three times with distilled water, while the aqueous layer was washed once with toluene. The combined organic layers were dried over anhydrous $MgSO_4$. Toluene was concentrated on a rotary vacuum evaporator and the resulting solid was recrystallized from ethanol. Yield 73.0g (84,0%). Mp = 101.0 – 102.0°C. MS(EI) m/z: 408(M+); 323; 219; 189; 161; 95; 63.

3'-fluoro-4'-(trifluoromethoxy)-[1,1'-biphenyl]-4-yl 4-(trifluoromethoxy)benzoate 8b

The synthesis was carried out in the same way as in the case of the synthesis of compound **7a**. The solid product was recrystallised from the ethanol. Then the compound was purified by column chromatography on silica gel using hexane as eluent and recrystallized from ethanol. Yield 2.0g (40%). Mp = 107.80°C.

3',5'-difluoro-4'-(trifluoromethoxy)-[1,1'-biphenyl]-4-yl 4-(trifluoromethoxy)benzoate 8c

The synthesis and purification procedure was carried out in the same way as in the case of the synthesis of compound **8b**. Yield 1.8g (60%). Mp = 80.20°C.

4-bromo-3-fluorophenyl-4-(trifluoromethoxy)benzoate 11
The synthesis was carried out in the same way as in the case of the synthesis of compound **9**. The solid product was recrystallised from the hexane. Yield 11.0g (76,0%). Mp = 105.9.

2,3'-difluoro-4'-(trifluoromethoxy)-[1,1'-biphenyl]-4-yl 4-(trifluoromethoxy)benzoate 13b
4-bromo-3-fluorophenyl-4-(trifluoromethoxy)benzoate **11** (3.0g – 8.0mmol), 2-(3-fluoro-4-(trifluoromethoxy)phenyl)-1,3,2-dioxaborinane **6b** (2.3g – 8.8mmol), potassium phosphate trihydrate $K_3PO_4 * 3H_2O$ (8.2g – 30.8mmol), tetrahydrofuran THF 50cm^3 were added to reaction vessel. Reaction was carried out under an N_2 atmosphere. The mixture was stirred and heated up to the boiling temperature and kept under this conditions for 15 minutes. Then it was cooled down to 50°C and ligand SPhos (1.0%mol) and catalyst $Pd(OAc)_2$ (0.3%mol) was added in one portion. The reaction mixture was heated under reflux for 1h and sample was analysed on GC-MS to check that all bromo-derivatives had reacted. The solid product was recrystallised from the methanol. Then the compound was purified by column chromatography on silica gel using hexane as eluent and recrystallized from methanol. Yield 1.5g (40.0%). Mp = 65.8°C.

2-fluoro-4'-(trifluoromethoxy)-[1,1'-biphenyl]-4-ol 12a
The synthesis was carried out in the same way as in the case of the synthesis of compound **13b**. Product was distilled under reduced pressure using a Büchi B-585 "kugelrohr" b.p 130 - 145°C (temperature on display) at 0.4mmHg. Yield 9.9g (67.2%).

2,3',5'-trifluoro-4'-(trifluoromethoxy)-[1,1'-biphenyl]-4-ol 12c
The synthesis was carried out in the same way as in the case of the synthesis of compound **13b**. Product was distilled under reduced pressure using a Büchi B-585 "kugelrohr" b.p 140 - 160°C (temperature on display) at 0.5mmHg. Yield 11.7g (84.0%).

3,3'-difluoro-4'-(trifluoromethoxy)-[1,1'-biphenyl]-4-ol 12d
The synthesis was carried out in the same way as in the case of the synthesis of compound **13b**. Product was distilled under reduced pressure using a Büchi B-585 "kugelrohr" b.p 145 - 170°C (temperature on display) at 0.4mmHg. Yield 12.8g (64.0%). Purity 98,6% (GC-FID).

2-fluoro-4'-(trifluoromethoxy)-[1,1'-biphenyl]-4-yl 4-(trifluoromethoxy)benzoate 13a
The synthesis was carried out in the same way as in the case of the synthesis of compound **8a**. The solid product was recrystallised from the ethanol. Then the compound was purified by column chromatography on silica gel using hexane as eluent and recrystallized from ethanol. Yield 2.3g (46.0%). Mp = 86.4°C.

2,3',5'-trifluoro-4'-(trifluoromethoxy)-[1,1'-biphenyl]-4-yl 4-(trifluoromethoxy)benzoate 13c
The synthesis and purification procedure was carried out in the same way as in the case of the synthesis of compound **13a**. Yield 1.5g (50%). Mp = 75.7°C.

3,3'-difluoro-4'-(trifluoromethoxy)-[1,1'-biphenyl]-4-yl 4-(trifluoromethoxy)benzoate 13d
The synthesis and purification procedure was carried out in the same way as in the case of the synthesis of compound **13a**. Yield 2.1g (42%). Mp = 82.7°C.

2-methyl-4-(4-(trifluoromethoxy)phenyl)but-3-yn-2-ol 16a
All the details are in the article[1]

4-(3-fluoro-4-(trifluoromethoxy)phenyl)-2-methylbut-3-yn-2-ol 16b
All the details are in the article[1]

4-(3,5-difluoro-4-(trifluoromethoxy)phenyl)-2-methylbut-3-yn-2-ol 16c
All the details are in the article[1]

4-(2-fluoro-4-(trifluoromethoxy)phenyl)-2-methylbut-3-yn-2-ol 16d
1-bromo-2-fluoro-4-(trifluoromethoxy)benzene **14d** (70.0g - 0.27mol), triethylamine TEA (37.4cm^3 - 0.27mol), 1.8-diazabicyclo[5.4.0]undec-7-ene DBU (40cm^3 - 0.27mol), $PdCl_2(PPh_3)_2$ (0.3mol%), CuI (0.1mol%) and 500cm^3 of toluene were added to reaction vessel. Reaction was carried out under an N_2 atmosphere. The mixture was stirred and heated up to the boiling temperature and kept under this conditions for 15 minutes. Then it was cooled down to 60°C and a solution of 2-methylbut-3-yn-2-ol **15** (29cm^3 - 0.297mol) in 60cm^3 of toluene was added dropwise and the reaction mixture was heated under reflux for 4h. When the reaction was finished resulting precipitate was filtered on a Büchner funnel. The filtrate was poured into a large amount of water and stirred for 15 minutes. Moved to a separating funnel, the layers were separated. The organic layer was washed three times with water, separated, dried with $MgSO_4$ and solvent evaporated. Product was distilled under reduced pressure b.p. 80.0°C at 0.4mmHg, yield 54.2g (77.0%). MS(EI) m/z: 262(M+); 247; 231; 177; 133; 69, 43.

1-ethynyl-4-(trifluoromethoxy)benzene 17a
All the details are in the article[1]

4-ethynyl-2-fluoro-1-(trifluoromethoxy)benzene 17b
All the details are in the article[1]

5-ethynyl-1,3-difluoro-2-(trifluoromethoxy)benzene 17c
All the details are in the article[1]

1-ethynyl-2-fluoro-4-(trifluoromethoxy)benzene 17d
4-(2-fluoro-4-(trifluoromethoxy)phenyl)-2-methylbut-3-yn-2-ol **16d** (54.2g – 0,207mol), catalytic amount of sodium hydride NaH (10mol%) and anhydrous toluene 300cm^3 were added to reaction vessel. The mixture was stirred under reflux while acetone was distilled off. When the reaction was completed, crude product was purified using column chromatography (silica gel and toluene as an eluent). Toluene was evaporated and crude product was distilled under reduced pressure b.p. 104.0°C at 200.0mmHg, yield 36.5g (86.4%).

4-((4-(trifluoromethoxy)phenyl)ethynyl)phenyl-4-(trifluoromethoxy)benzoate 18a
The synthesis was carried out in the same way as in the case of the synthesis of compound **16d**. The solid product was recrystallised from the ethanol. Then the compound was purified by column chromatography on silica gel using hot hexane (40°C) as eluent and recrystallized from ethanol. Yield 4.2g (52.4%). Mp = 114.1°C.

4-((3-fluoro-4-(trifluoromethoxy)phenyl)ethynyl)phenyl-4-(trifluoromethoxy)benzoate 18b

The synthesis and purification procedure was carried out in the same way as in the case of the synthesis of compound **18a**. Yield 3.9g (46.8%). Mp = 87.5°C.

4-((3,5-difluoro-4-(trifluoromethoxy)phenyl)ethynyl)phenyl-4-(trifluoromethoxy)benzoate 18c

The synthesis and purification procedure was carried out in the same way as in the case of the synthesis of compound **18a**. Yield 3.8g (44.0%). Mp = 74.3°C.

4-((2-fluoro-4-(trifluoromethoxy)phenyl)ethynyl)phenyl-4-(trifluoromethoxy)benzoate 18d

The synthesis and purification procedure was carried out in the same way as in the case of the synthesis of compound **18a**. Yield 5.1g (61.3%). Mp = 71.1°C.

3-fluoro-4-(trifluoromethoxy)phenol 19

2-(3-fluoro-4-(trifluoromethoxy)phenyl)-1,3,2-dioxaborinane **6b** (10.0g – 38.0mmol) and 150cm^3 acetone were added to reaction vessel. While stirring, an aqueous solution of OXONE (30cm^3) was added in one portion. A very strong exothermic effect was observed. The temperature increase to near the boiling point. Then it was stirred for 1h. When the reaction was completed, it was poured into 100 cm^3 of H_2O and extracted with CH_2Cl_2. The organic layer was washed three times with water, separated, dried with $MgSO_4$ and solvent evaporated. Then the compound was purified by column chromatography on silica gel using hexane as eluent. The crude product was used for the next steps.

3-fluoro-4-(trifluoromethoxy)phenyl 4-(trifluoromethoxy)benzoate 20

The synthesis was carried out in the same way as in the case of the synthesis of compound **8a**. Then the compound was purified by column chromatography on silica gel using hexane as eluent. The solid product was recrystallised from the ethanol. Due to the fact the compound did not crystallize at -15°C, it was decided to put a magnetic stirrer in the flask and force the crystallization at -78°C (acetone - dry ice bath). The obtained crystal was immediately filtered off. Yield 5.4g (37.0%). Mp = 44.8°C.

Refractive indices data

Table S1. Measured refractive indices (n_e and n_o) of **8b** at λ=443, 636, and 1550 nm, and at different temperatures.

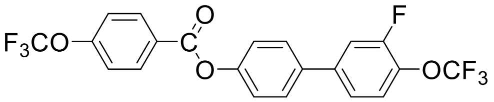

T (°C)	λ (nm)					
	443nm		636nm		1550nm	
	n_o	n_e	n_o	n_e	n_o	n_e
110	1.46578	1.66896	1.44281	1.62942	1.42860	1.58634
115	1.46574	1.66201	1.44320	1.62456	1.42990	1.58023
120	1.46578	1.65764	1.44419	1.61939	1.43001	1.57568
125	1.46712	1.65111	1.44499	1.61201	1.43050	1.56801
130	1.46805	1.6433	1.44627	1.60517	1.43082	1.56151
135	1.47013	1.63423	1.44825	1.59621	1.43264	1.55402
140	1.50660		1.47590		1.45880	
145	1.50460		1.47496		1.45489	

Table S2. Measured refractive indices (n_e and n_o) of **13b** at λ=443, 636, and 1550 nm, and at different temperatures.

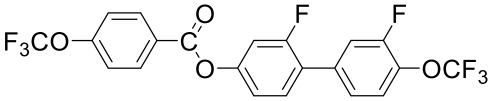

T (°C)	λ (nm)					
	443nm		636nm		1550nm	
	n_o	n_e	n_o	n_e	n_o	n_e
70	1.46397	1.62339	1.44389	1.60963	1.42880	1.57530
75	1.46379	1.62022	1.44380	1.60578	1.42850	1.57355
80	1.46370	1.61750	1.44389	1.60096	1.42820	1.56848
85	1.46371	1.61222	1.44370	1.59500	1.42830	1.56431
90	1.46395	1.60775	1.44409	1.59031	1.42820	1.55806
95	1.46450	1.60125	1.44469	1.58533	1.42840	1.55440
100	1.46852	1.59755	1.44568	1.57751	1.42951	1.54638
105	1.46985	1.59035	1.44805	1.56822	1.43133	1.53758
110	1.50745		1.48315		1.46859	
115	1.50422		1.48156		1.46158	

Table S3. Measured refractive indices (n_e and n_o) of **8c** at λ=443, 636, and 1550 nm, and at different temperatures.

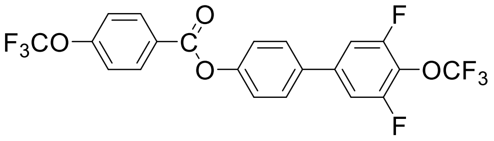

T (ºC)	λ (nm)					
	443nm		636nm		1550nm	
	n_o	n_e	n_o	n_e	n_o	n_e
85	1.47286	1.60180	1.45083	1.59232	1.43425	1.55509
90	1.47468	1.59440	1.45192	1.58132	1.43516	1.54361
95	1.47833	1.58760	1.45489	1.57285	1.43677	1.53415
100	1.51650		1.49024		1.46500	
105	1.51370		1.48844		1.46223	

Table S4. Measured refractive indices (n_e and n_o) of **13c** at λ=443, 636, and 1550 nm, and at different temperatures.

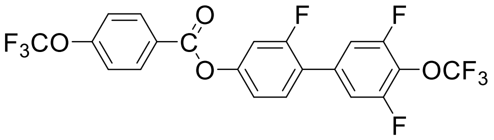

T (°C)	λ (nm)					
	443nm		636nm		1550nm	
	n_o	n_e	n_o	n_e	n_o	n_e
75	1.47086	1.59230	1.44914	1.56735	1.43244	1.53857
80	1.47532	1.58233	1.45222	1.55922	1.43455	1.53156
85	1.52420		1.48575		1.46562	
90	1.51215		1.48405		1.46340	

Table S5. Measured refractive indices (n_e and n_o) of **18b** at λ=443, 636, and 1550 nm, and at different temperatures.

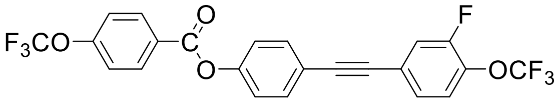

T (°C)	λ (nm)					
	443nm		636nm		1550nm	
	n_o	n_e	n_o	n_e	n_o	n_e
95	1.47188	1.73430	1.43998	1.67804	1.42233	1.62822
100	1.47244	1.73151	1.43995	1.67315	1.42254	1.62424
105	1.47115	1.72670	1.43994	1.66691	1.42235	1.62019
110	1.47114	1.72244	1.43955	1.66174	1.42244	1.61624
115	1.47154	1.71840	1.43977	1.65775	1.42248	1.61091
120	1.47080	1.71211	1.43988	1.65300	1.42304	1.60610
125	1.47077	1.70744	1.43968	1.64780	1.42288	1.60150
130	1.46977	1.70125	1.43954	1.64110	1.42110	1.59540
135	1.46819	1.69258	1.43964	1.63640	1.42094	1.58987
140	1.46757	1.68644	1.43988	1.63122	1.42200	1.58440
145	1.46801	1.67856	1.44022	1.62518	1.42280	1.57980
150	1.46811	1.66944	1.44112	1.61724	1.42288	1.57322
155	1.46935	1.66211	1.44241	1.60954	1.42306	1.56640
160	1.47199	1.65411	1.44355	1.60155	1.42444	1.55922
165	1.47505	1.64344	1.44557	1.59425	1.42771	1.55224
170	1.47911	1.63348	1.44740	1.58480	1.43022	1.54256
175	1.52452		1.48834		1.46148	
180	1.52211		1.48588		1.45987	

Table S6. Measured refractive indices (n_e and n_o) of **13c** at λ=443, 636, and 1550 nm, and at different temperatures.

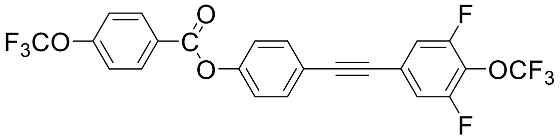

T (°C)	λ (nm)					
	443nm		636nm		1550nm	
	n_o	n_e	n_o	n_e	n_o	n_e
80	1.46790	1.71432	1.44548	1.67615	1.42780	1.62427
85	1.46710	1.70901	1.44520	1.67020	1.42760	1.62222
90	1.46700	1.70607	1.44508	1.66731	1.42719	1.61998
95	1.46660	1.70422	1.44499	1.66123	1.42712	1.61750
100	1.46800	1.70002	1.44449	1.65877	1.42709	1.61280
105	1.46770	1.69680	1.44460	1.65231	1.42680	1.60823
110	1.46669	1.69211	1.44429	1.64830	1.42638	1.60293
115	1.46800	1.68780	1.44460	1.64120	1.42640	1.59546
120	1.46990	1.68345	1.44488	1.63659	1.42638	1.58957
125	1.47055	1.67987	1.44555	1.62801	1.42654	1.58303
130	1.47123	1.67155	1.44647	1.62124	1.42689	1.57655
135	1.47150	1.66423	1.44900	1.61405	1.42801	1.56901
140	1.47342	1.65711	1.45390	1.60705	1.42961	1.56104
145	1.53522		1.49980		1.46999	
150	1.53611		1.49743		1.46945	

Table S7. Weight composition and phase transition temperatures of nematic mixture.

multicomponent mixture composition	weight %	
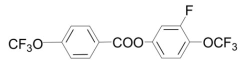	32.0	
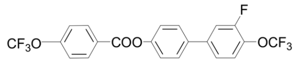	3.8	
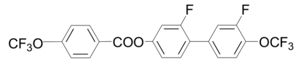	18.4	
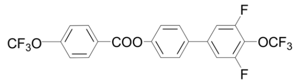	11.0	Cr -10 N 52.0-54.9 Iso
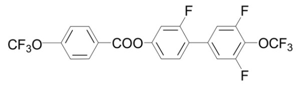	3.1	
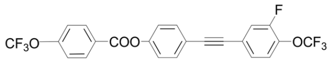	12.6	
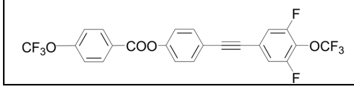	19.1	

Table S8. Measured refractive indices (n_e and n_o) of multicomponent nematic mixture at λ=443, 636, and 1550 nm, and at different temperatures.

T (°C)	λ (nm)					
	443nm		636nm		1550nm	
	n_o	n_e	n_o	n_e	n_o	n_e
20	1.47755	1.65001	1.45610	1.60222	1.43655	1.57101
25	1.47787	1.64870	1.45592	1.59730	1.43642	1.56681
30	1.47805	1.64422	1.45601	1.59426	1.43626	1.56421
35	1.47877	1.63829	1.45592	1.58808	1.43608	1.56069
40	1.47950	1.63096	1.45692	1.58258	1.43627	1.55382
45	1.48548	1.62494	1.45810	1.57548	1.43781	1.54762
50	1.48548	1.61089	1.46191	1.56524	1.44145	1.53724
55	1.52664		1.49470		1.47750	
60	1.52452		1.49362		1.47553	
65	1.52272		1.49172		1.47363	

Differential scanning calorimetry (DSC) graphs

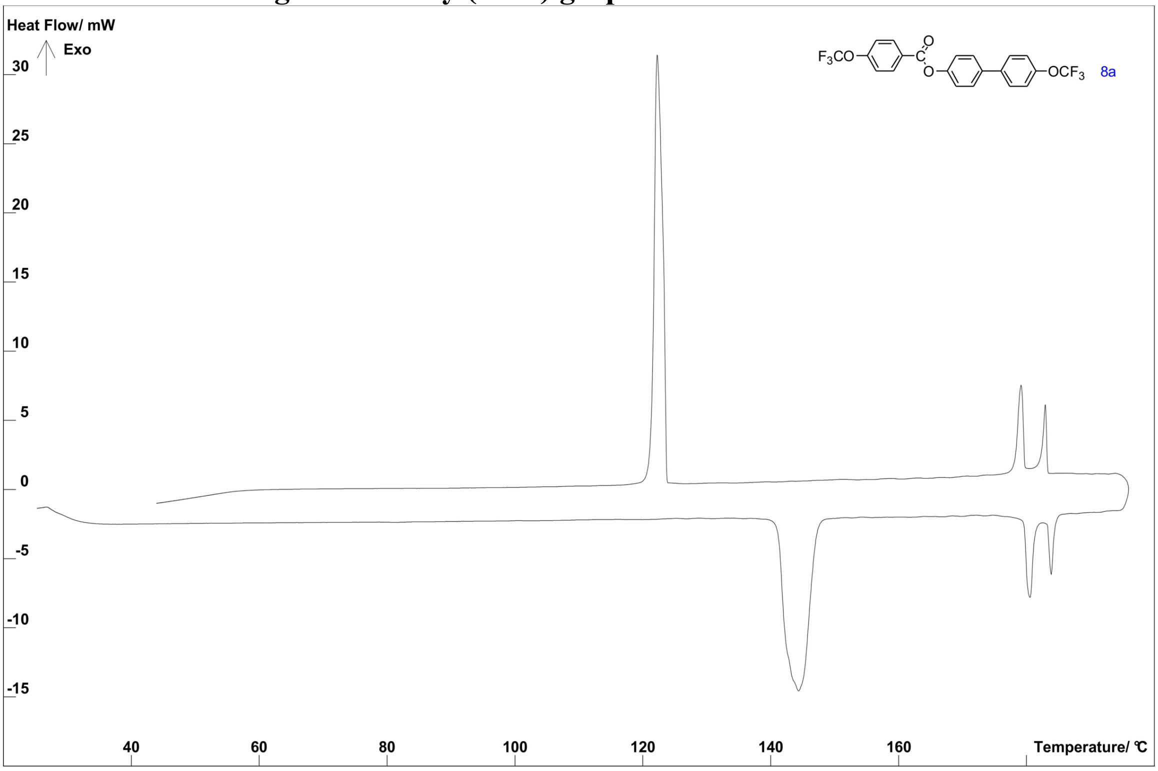

Figure S1. DSC spectrum for compound of **8a**

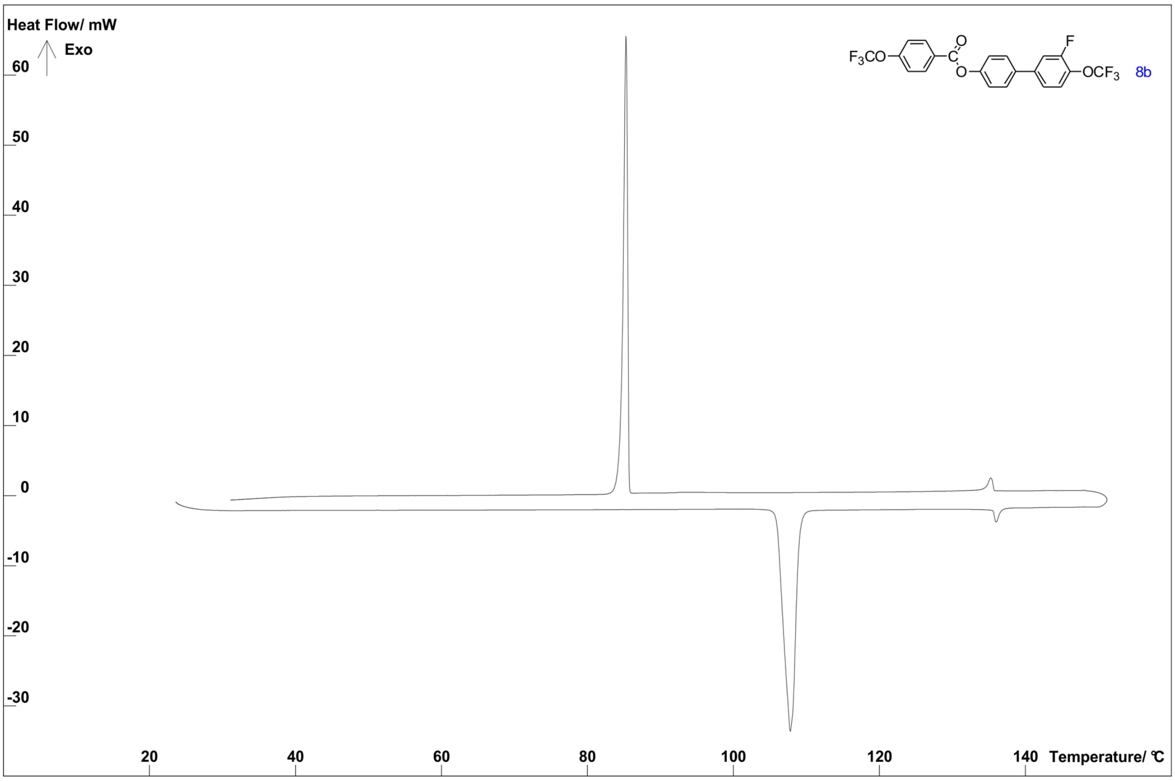

Figure S2. DSC spectrum for compound of **8b**

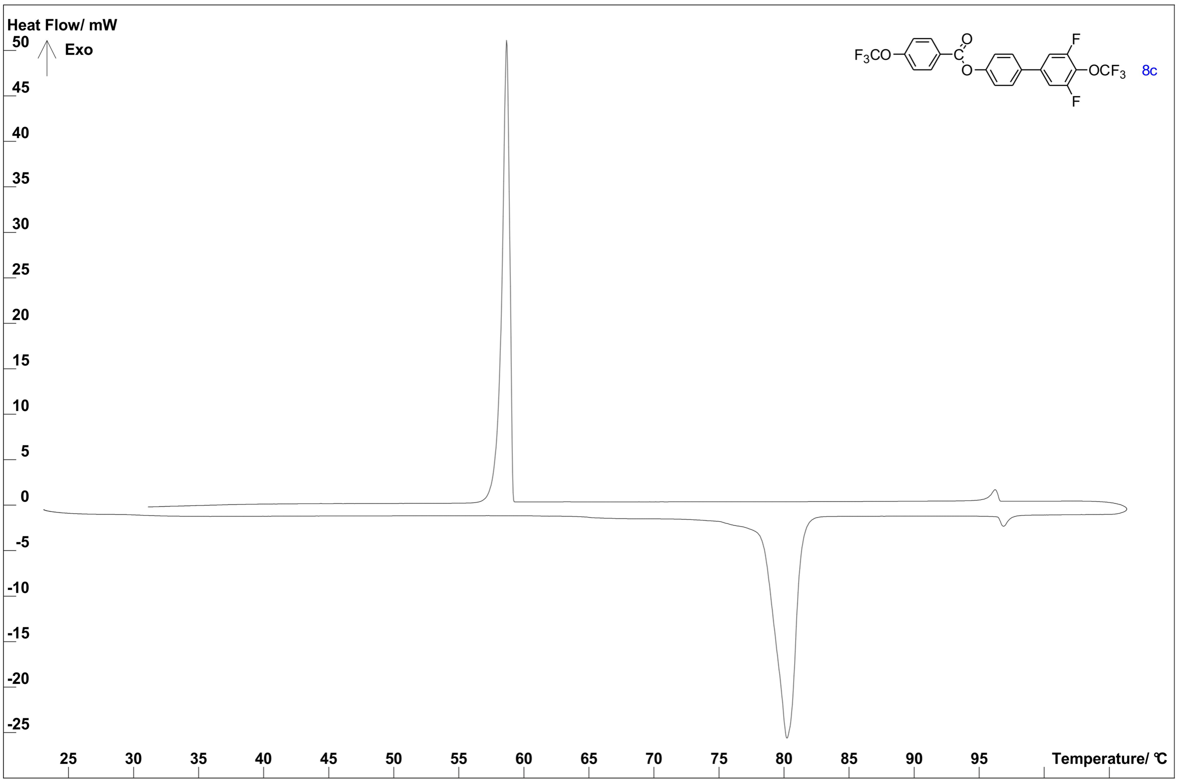

Figure S3. DSC spectrum for compound of **8c**

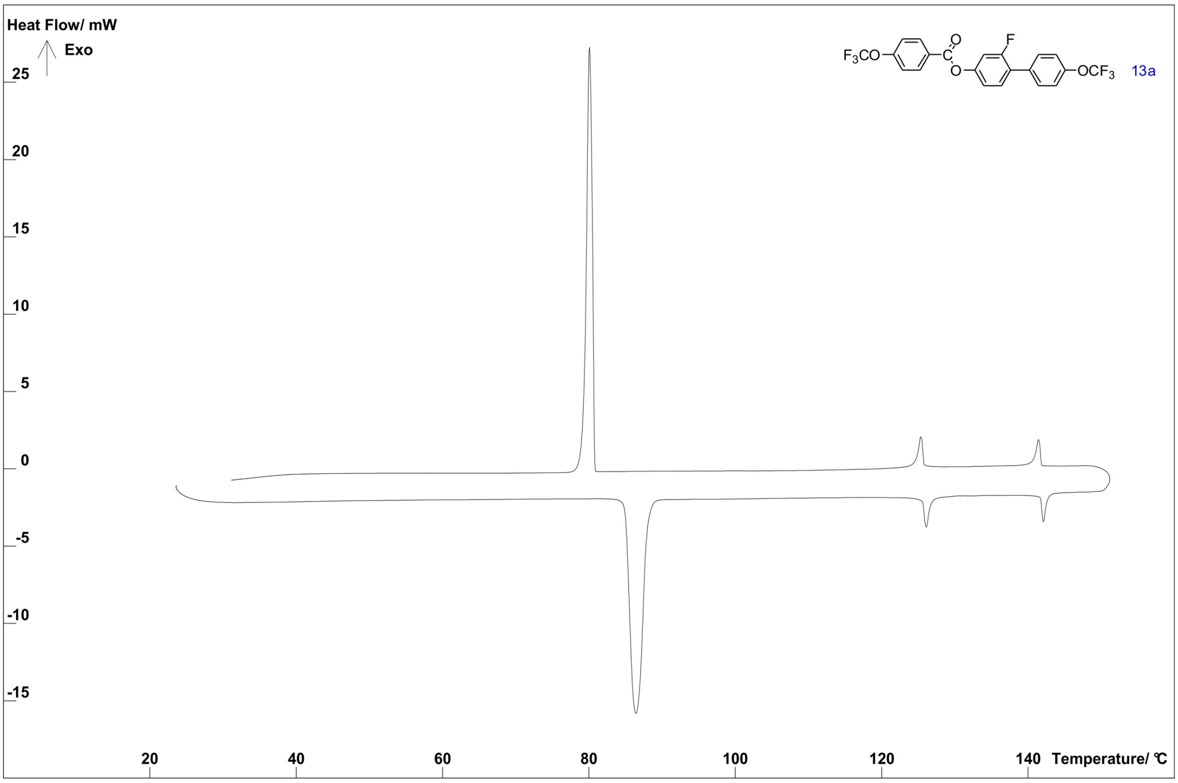

Figure S4. DSC spectrum for compound of **13a**

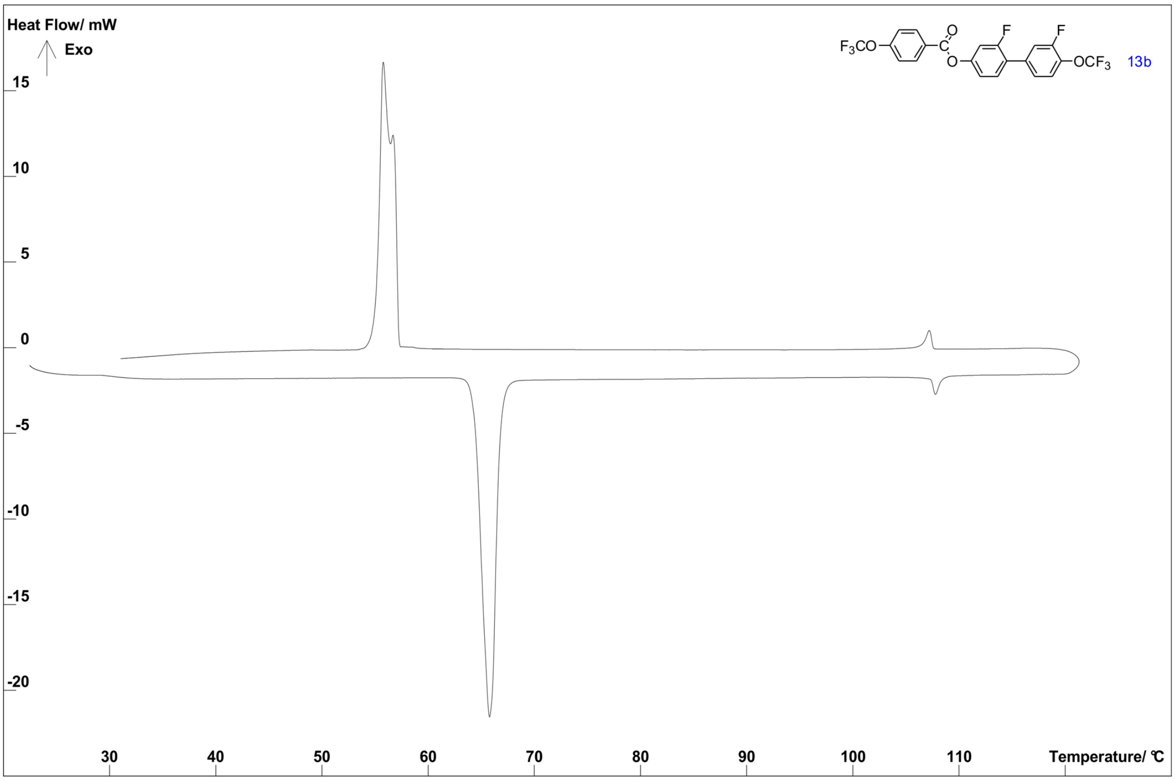

Figure S5. DSC spectrum for compound of **13b**

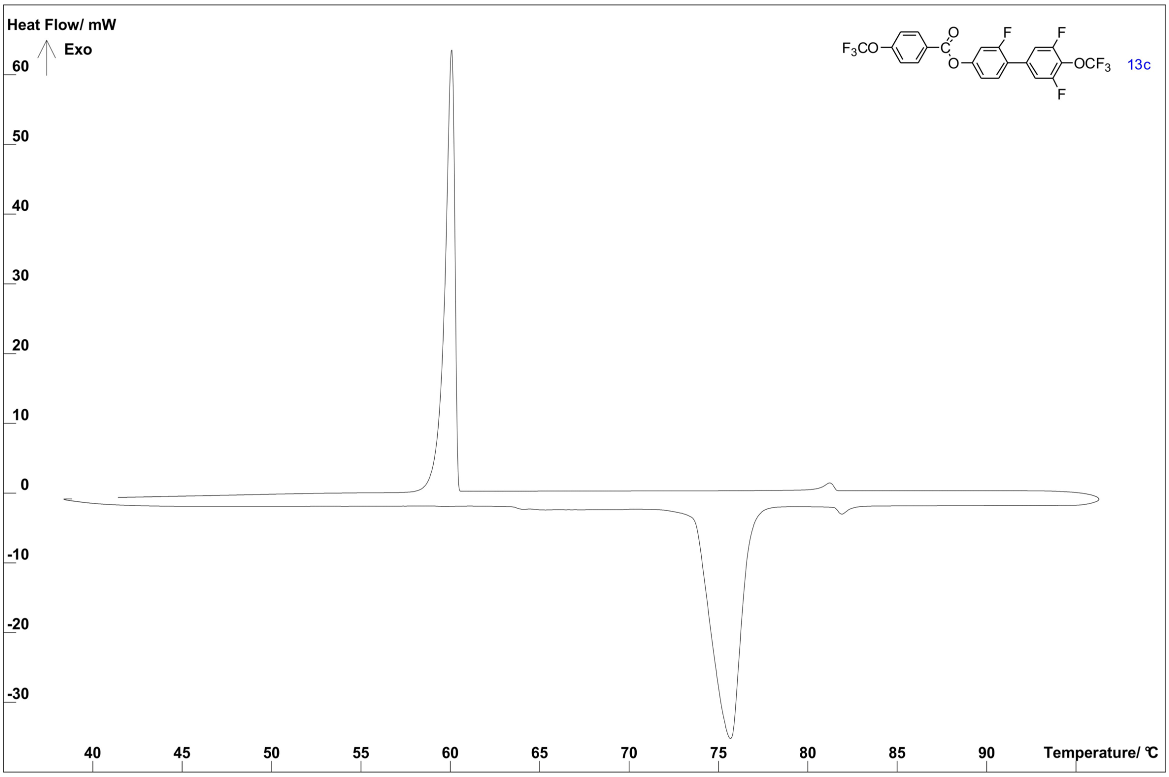

Figure S6. DSC spectrum for compound of **13c**

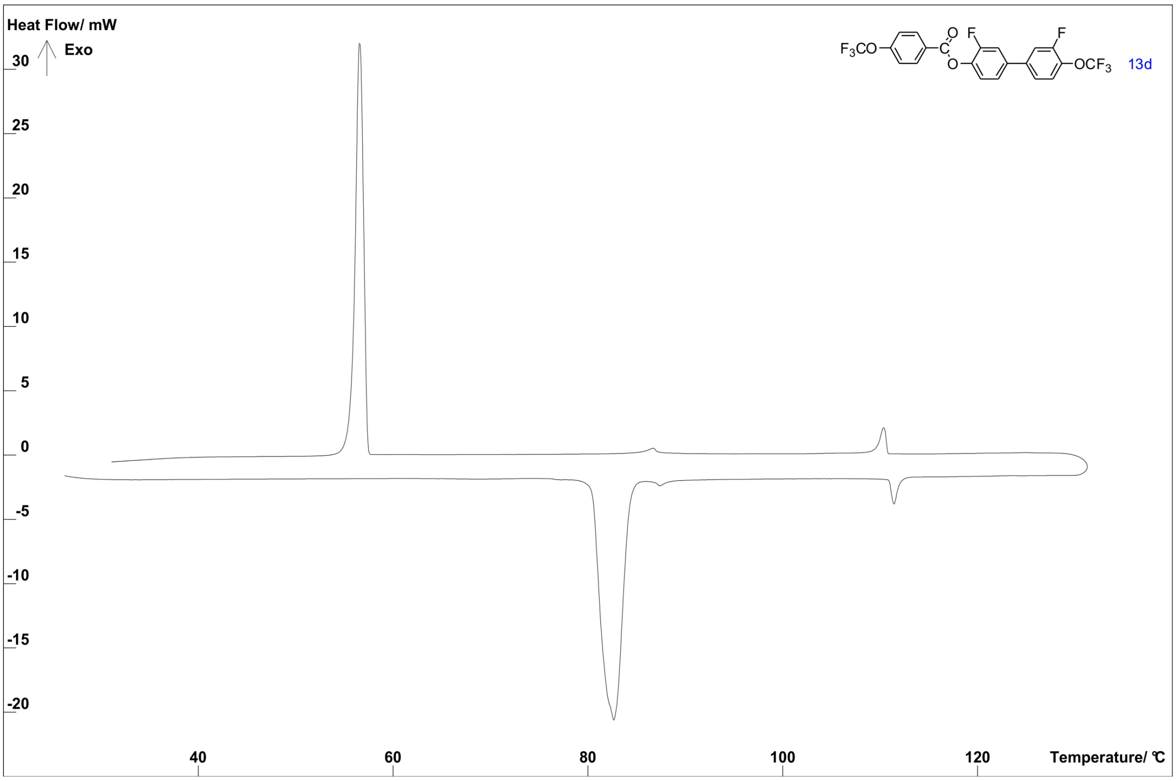

Figure S7. DSC spectrum for compound of **13d**

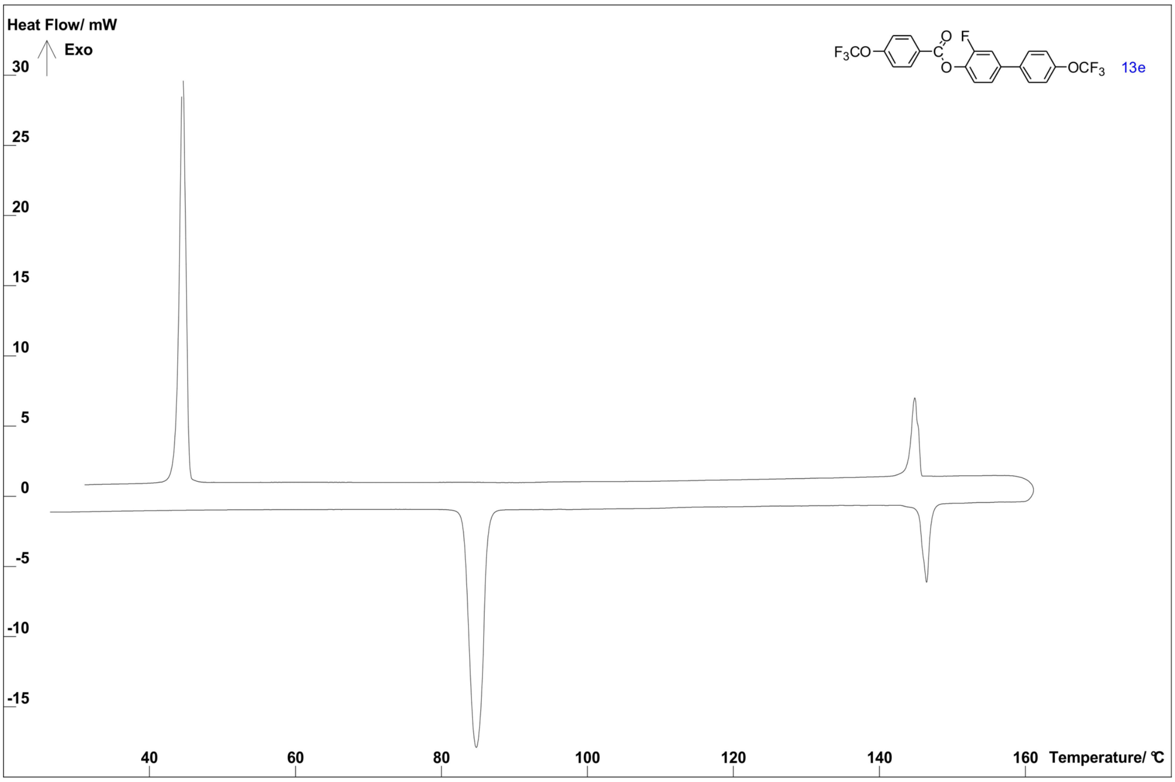

Figure S8. DSC spectrum for compound of **13e**

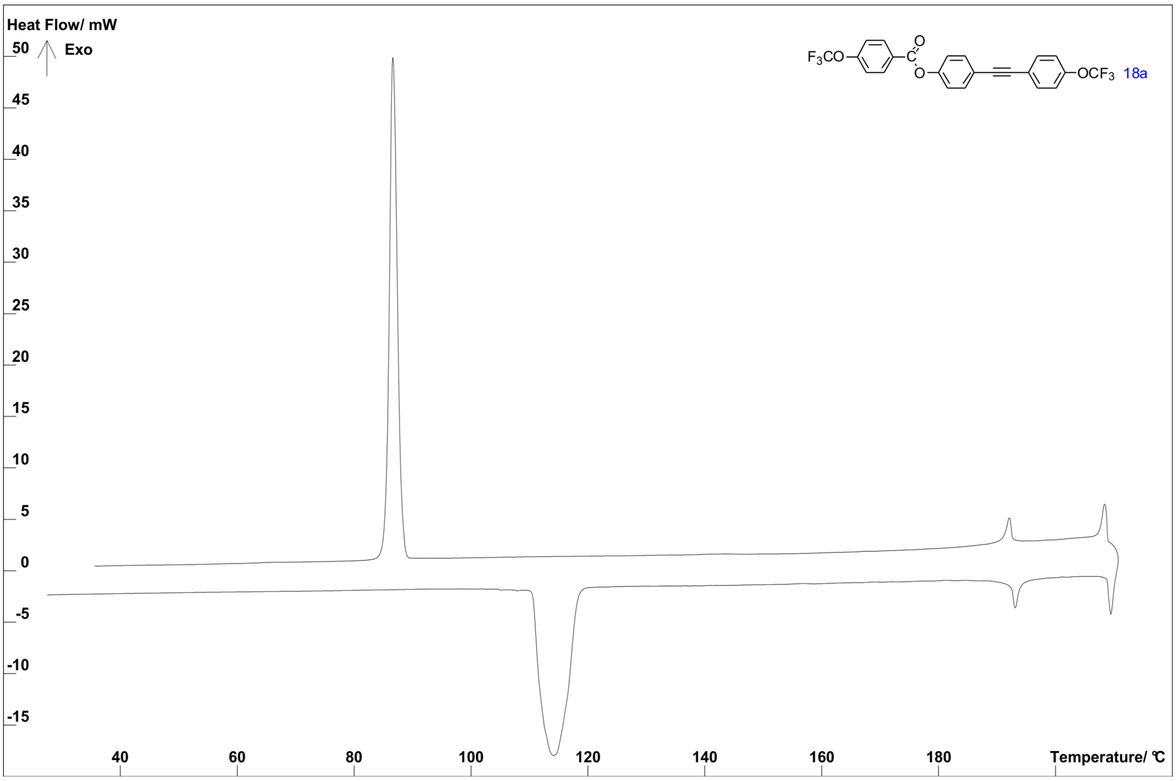

Figure S9. DSC spectrum for compound of **18a**

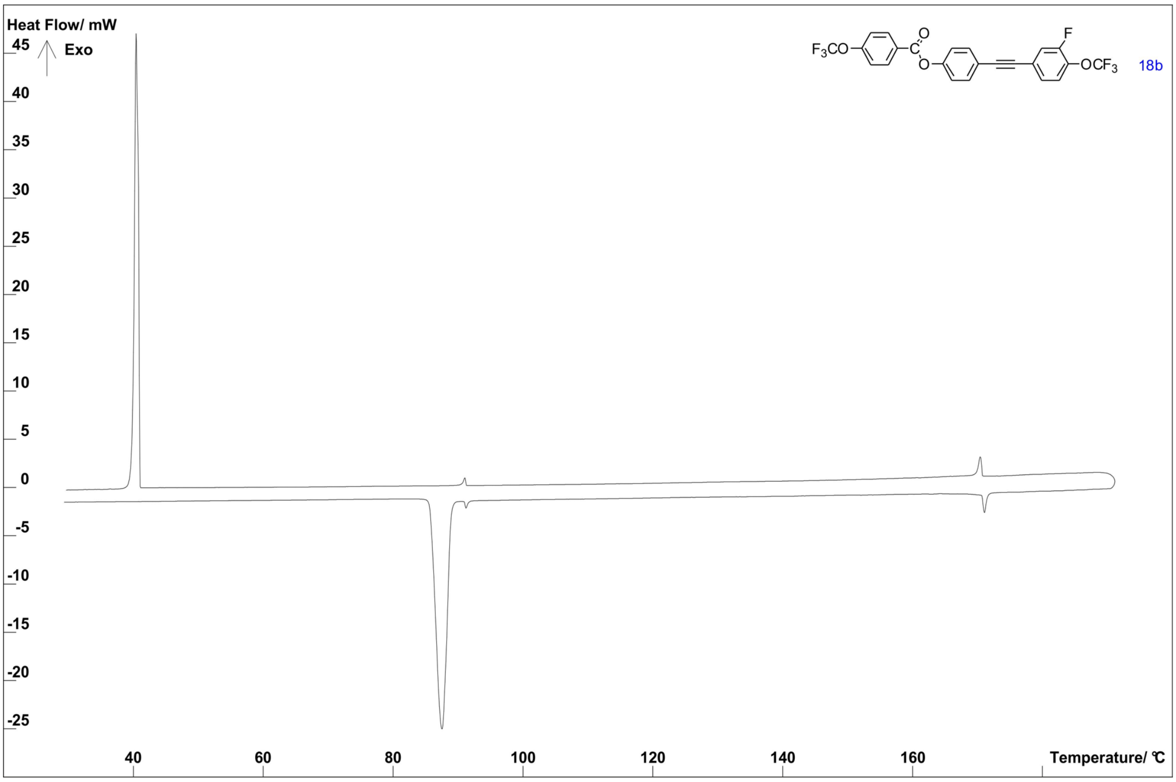

Figure S10. DSC spectrum for compound of **18b**

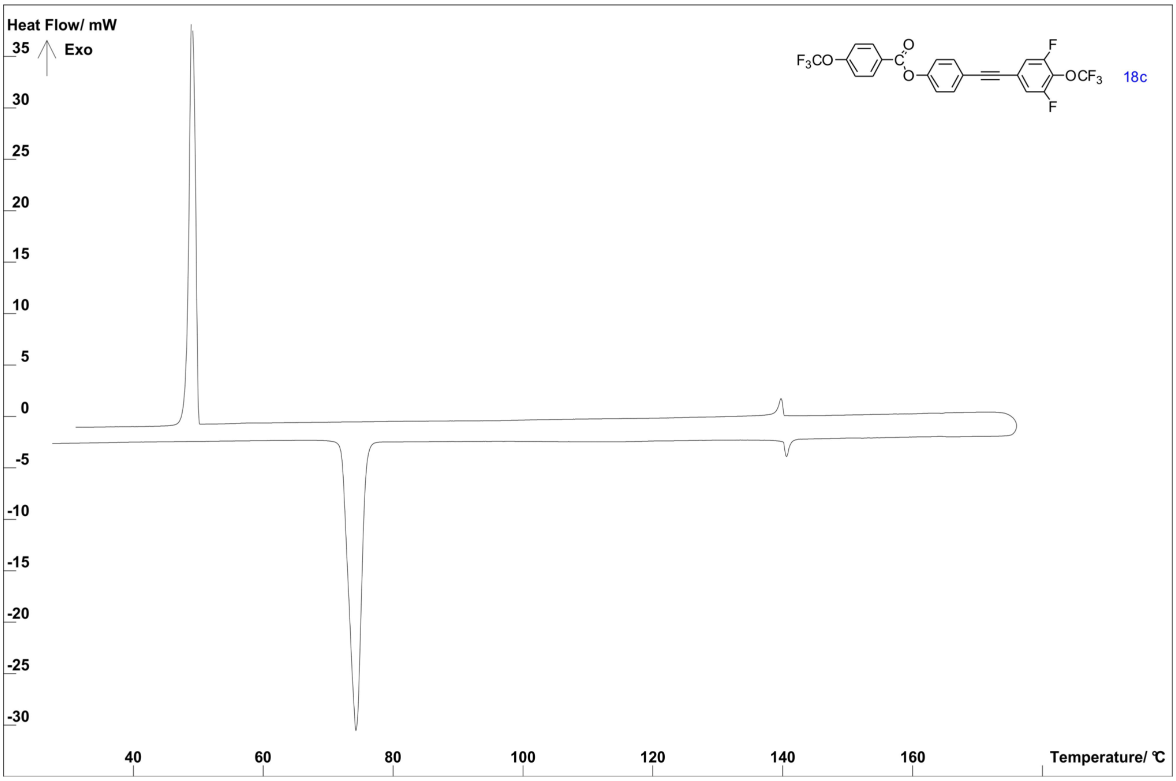

Figure S11. DSC spectrum for compound of **18c**

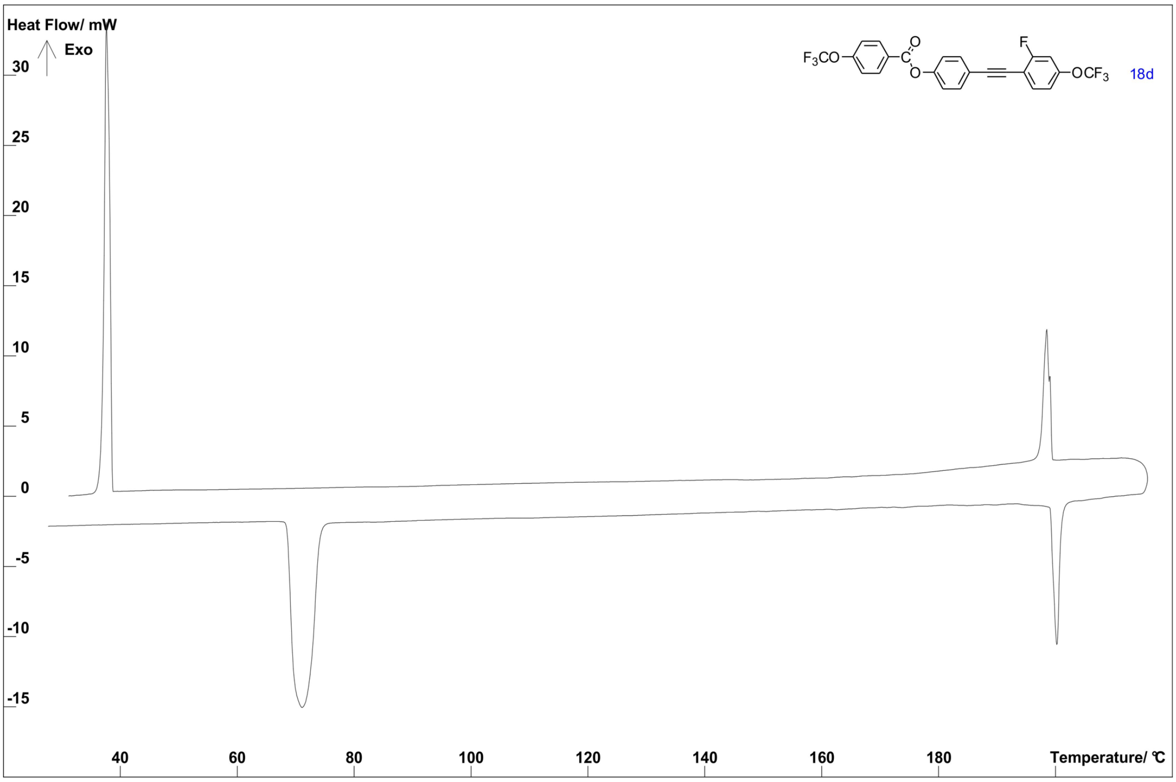

Figure S12. DSC spectrum for compound of **18d**

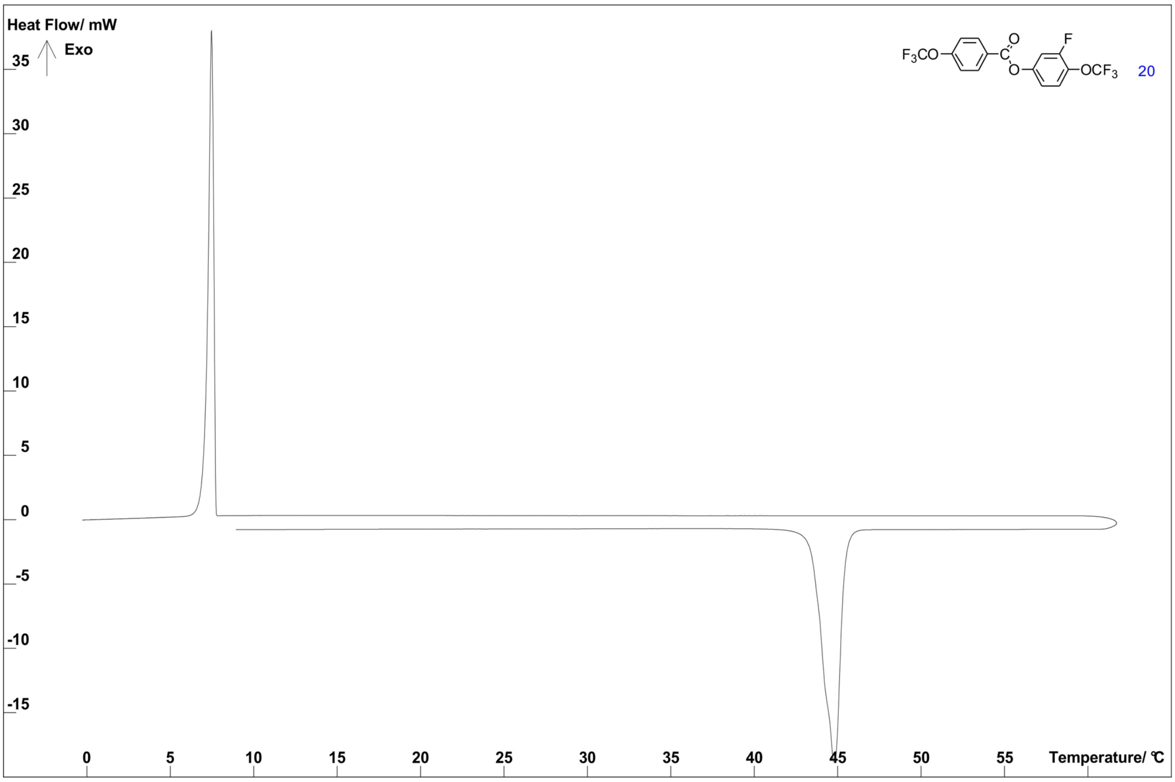

Figure S13. DSC spectrum for compound of **20**

Mass Spectrum graphs MS(EI)

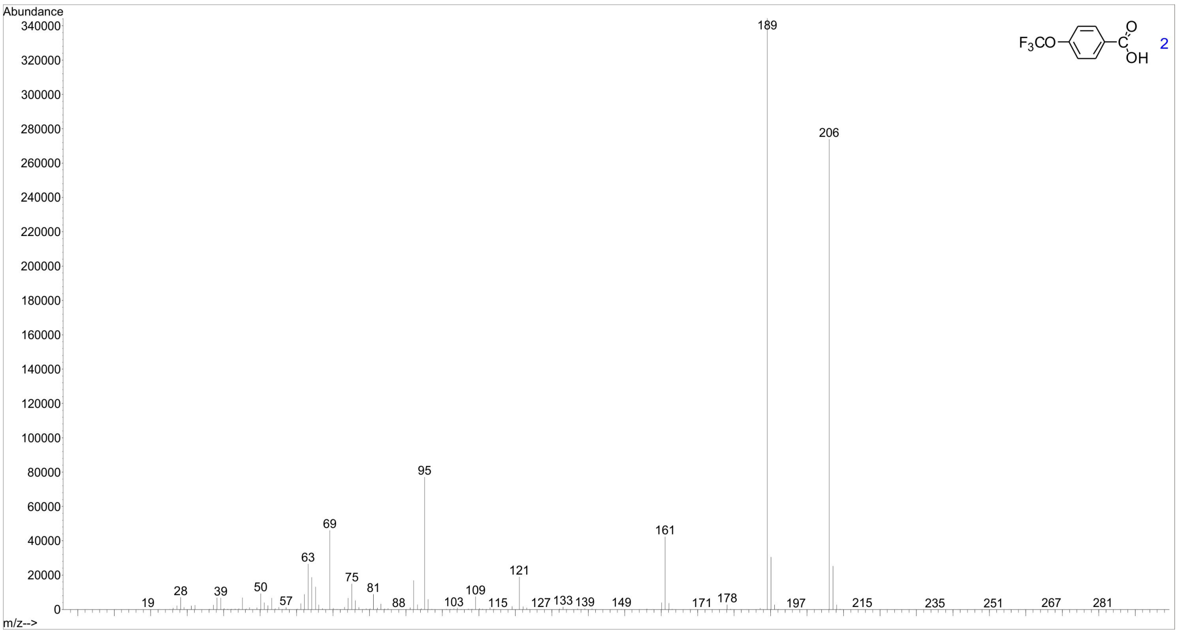

Figure S14. MS spectrum for compound of **2**

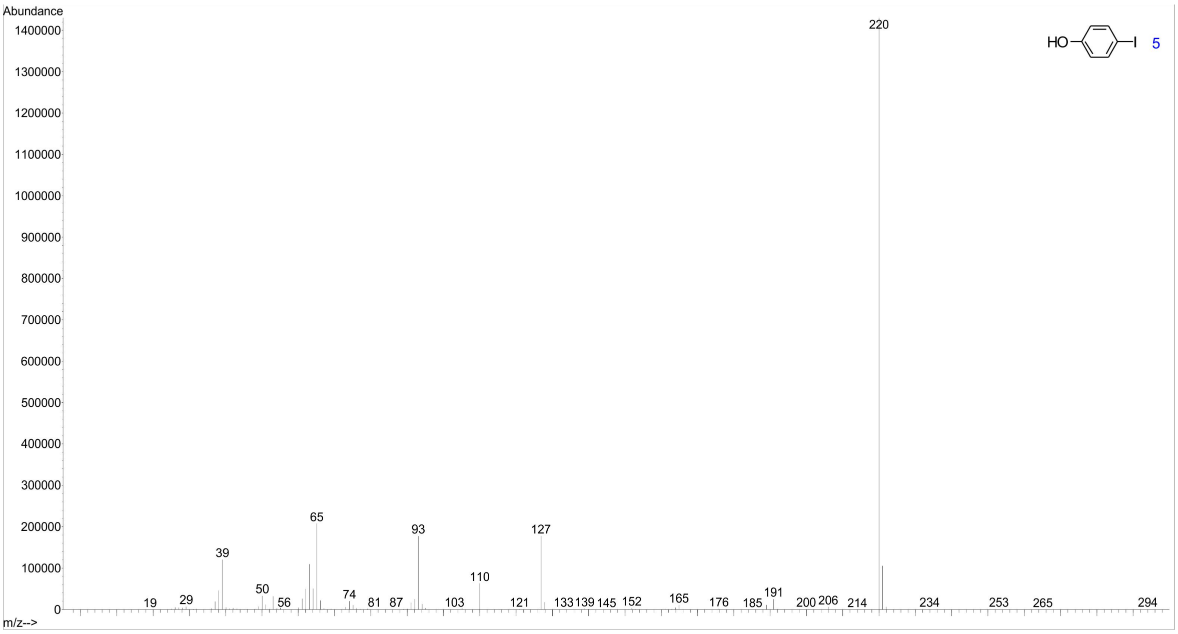

Figure S15. MS spectrum for compound of **5**

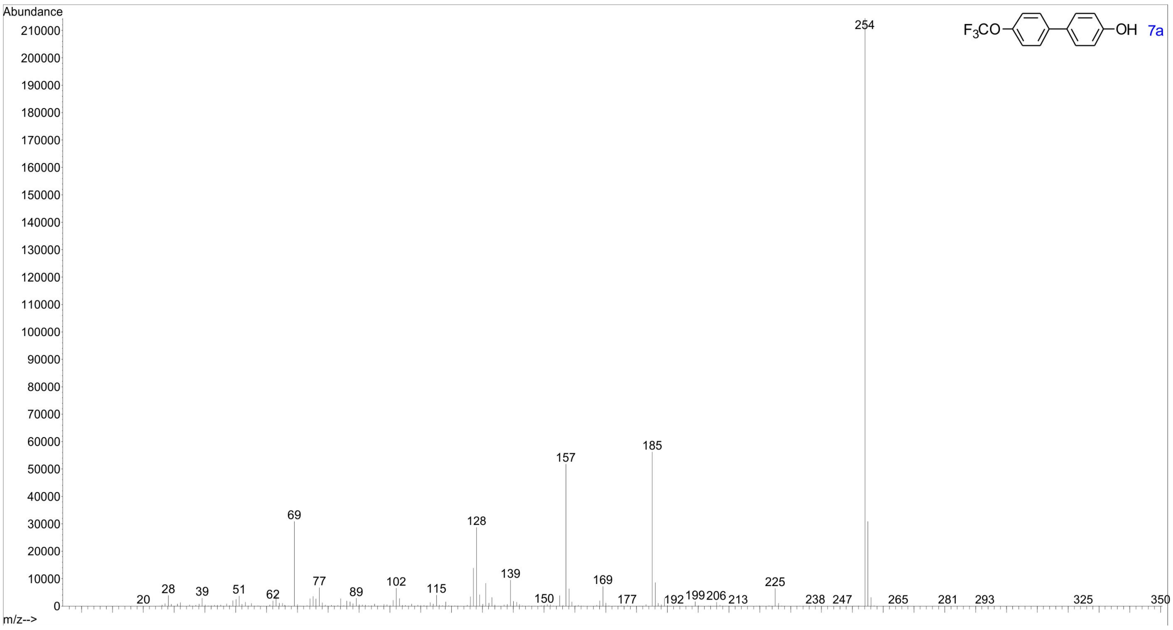

Figure S16. MS spectrum for compound of **7a**

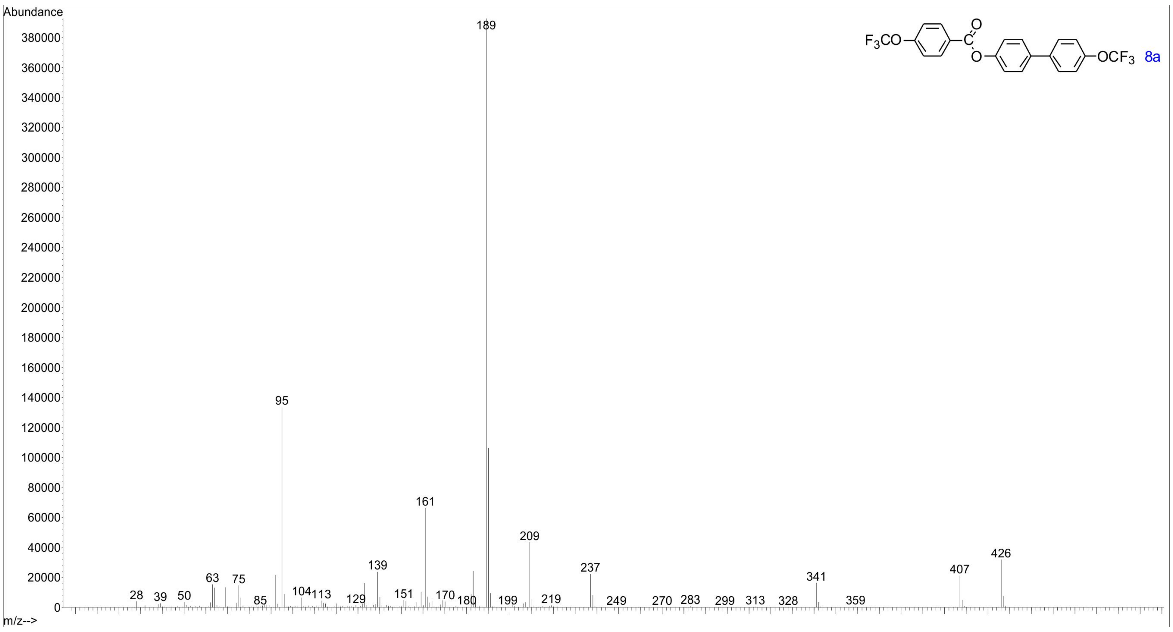

Figure S17. MS spectrum for compound of **8a**

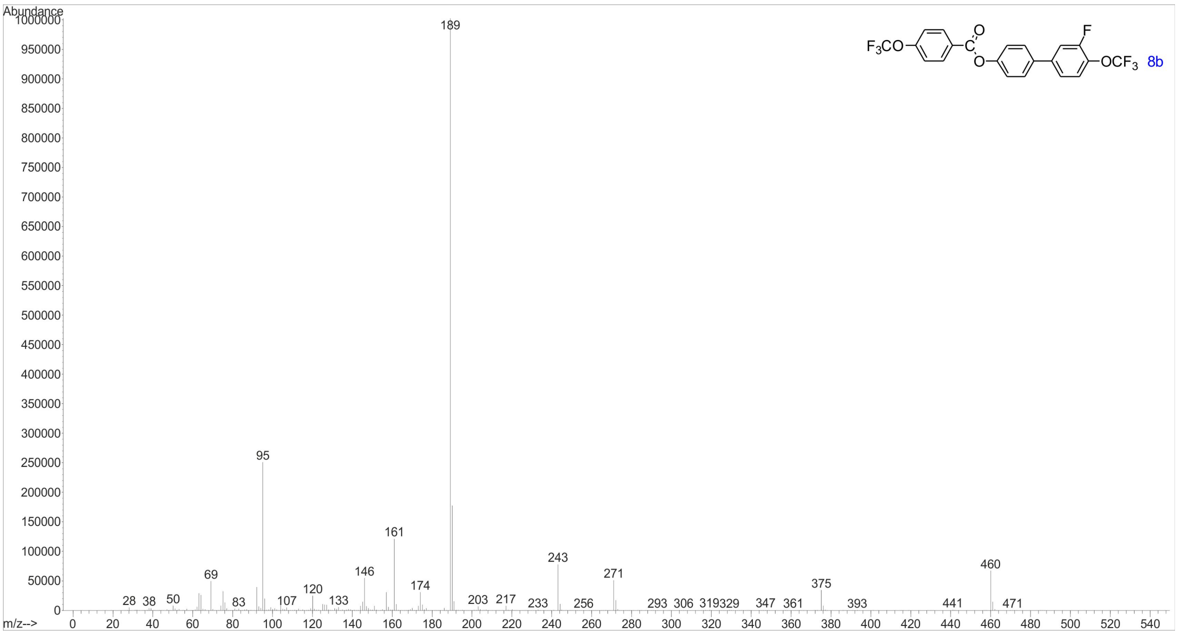

Figure S18. MS spectrum for compound of **8b**

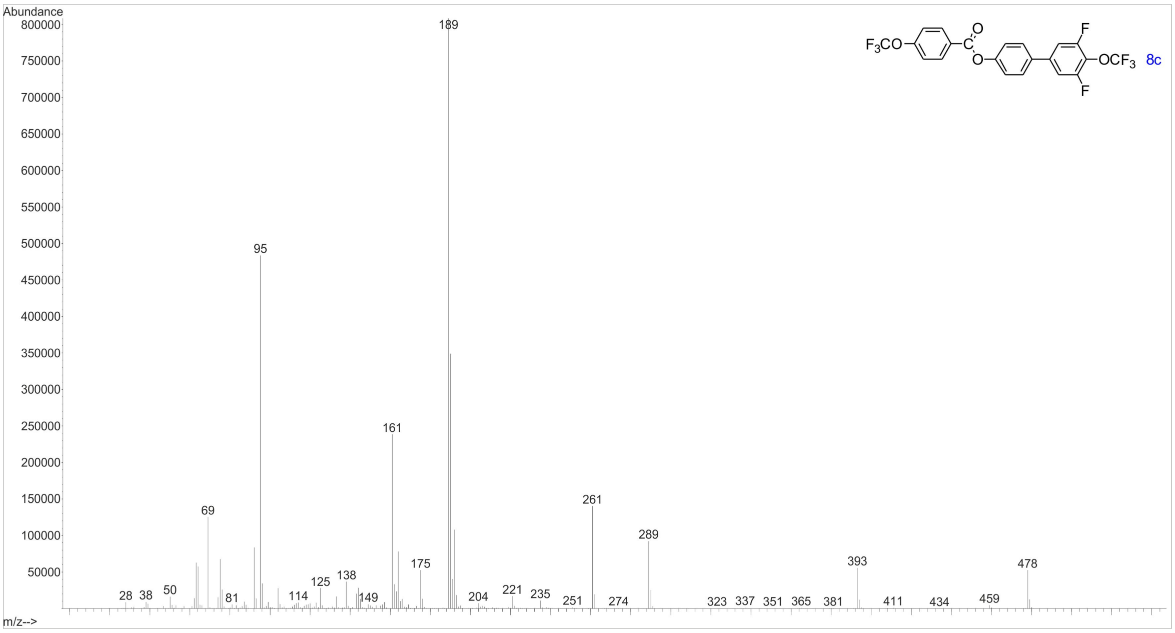

Figure S19. MS spectrum for compound of **8c**

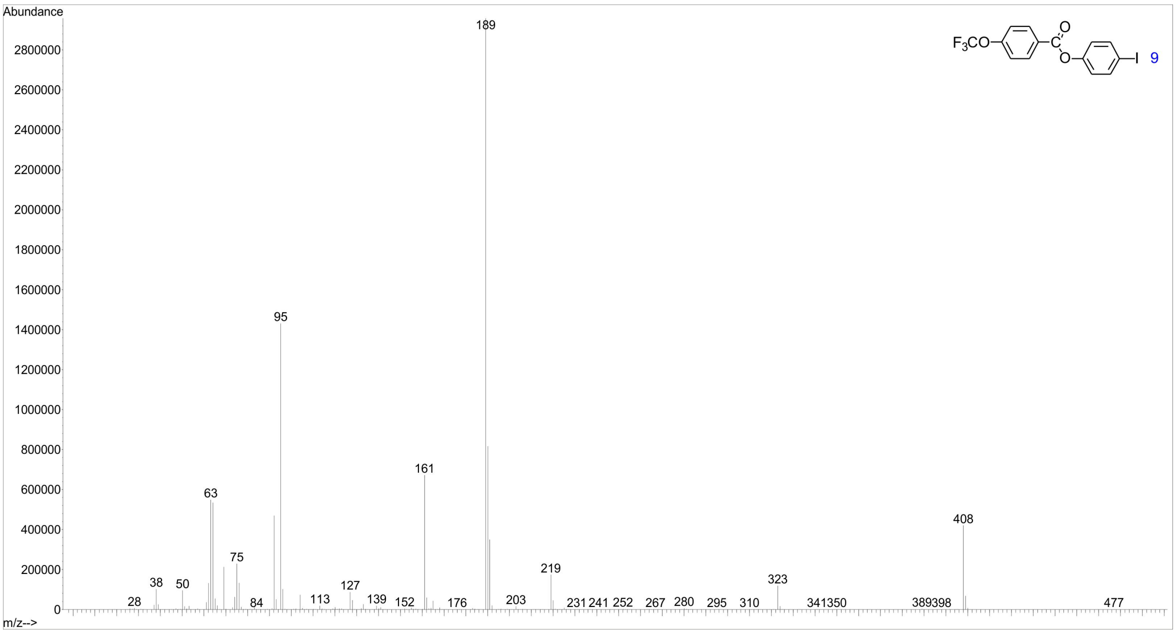

Figure S20. MS spectrum for compound of **9**

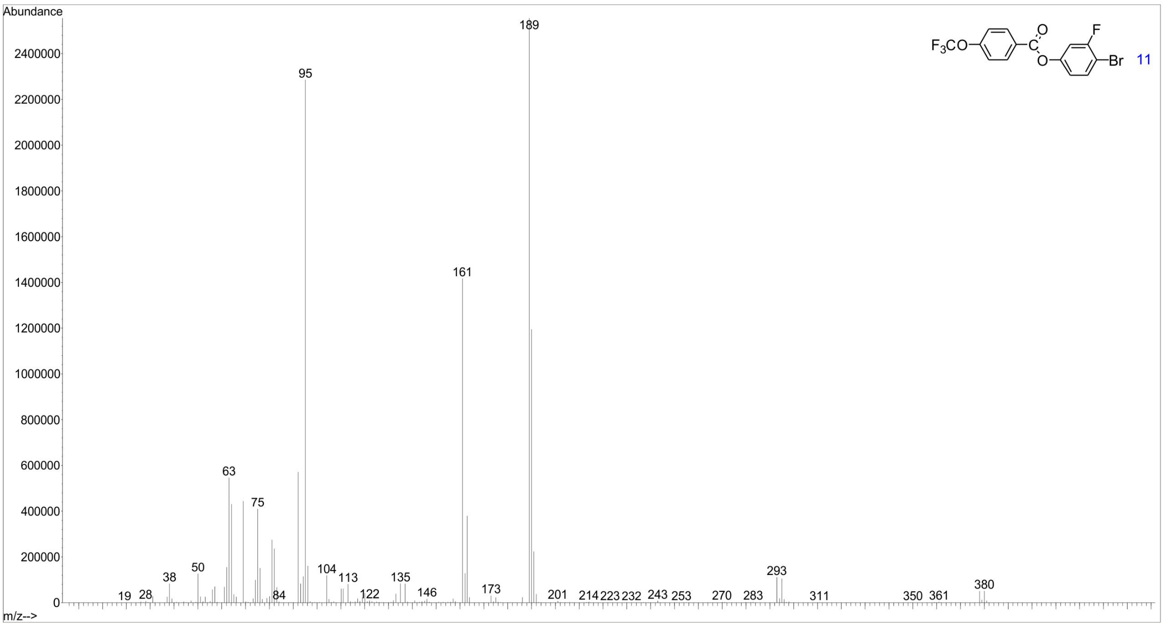

Figure S21. MS spectrum for compound of **11**

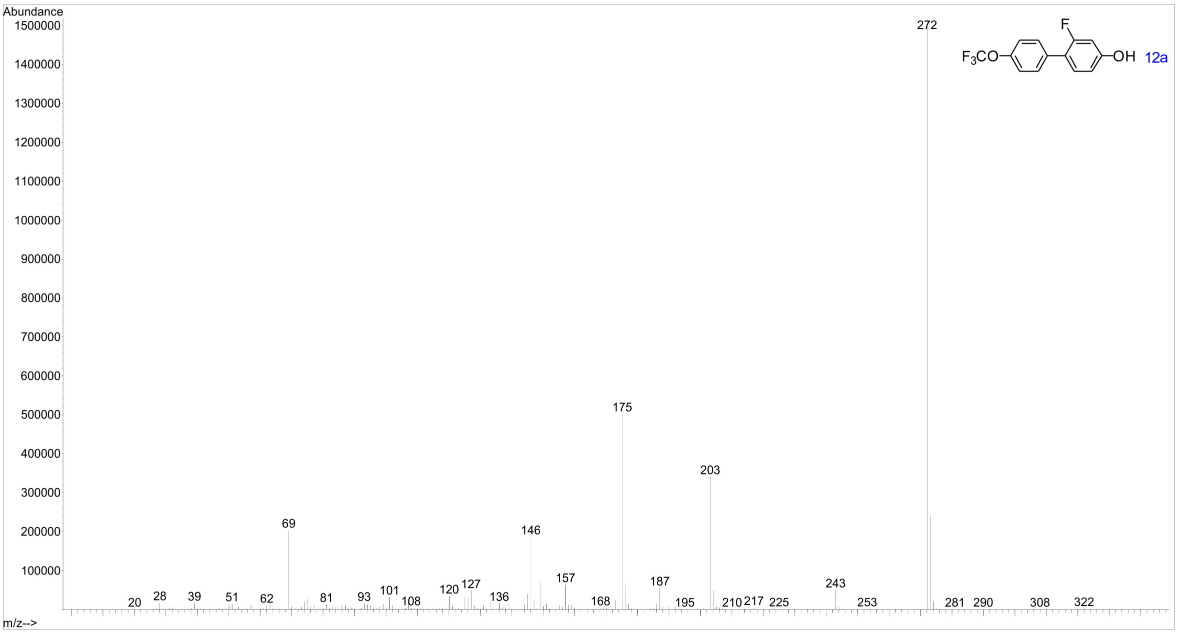

Figure S22. MS spectrum for compound of **12a**

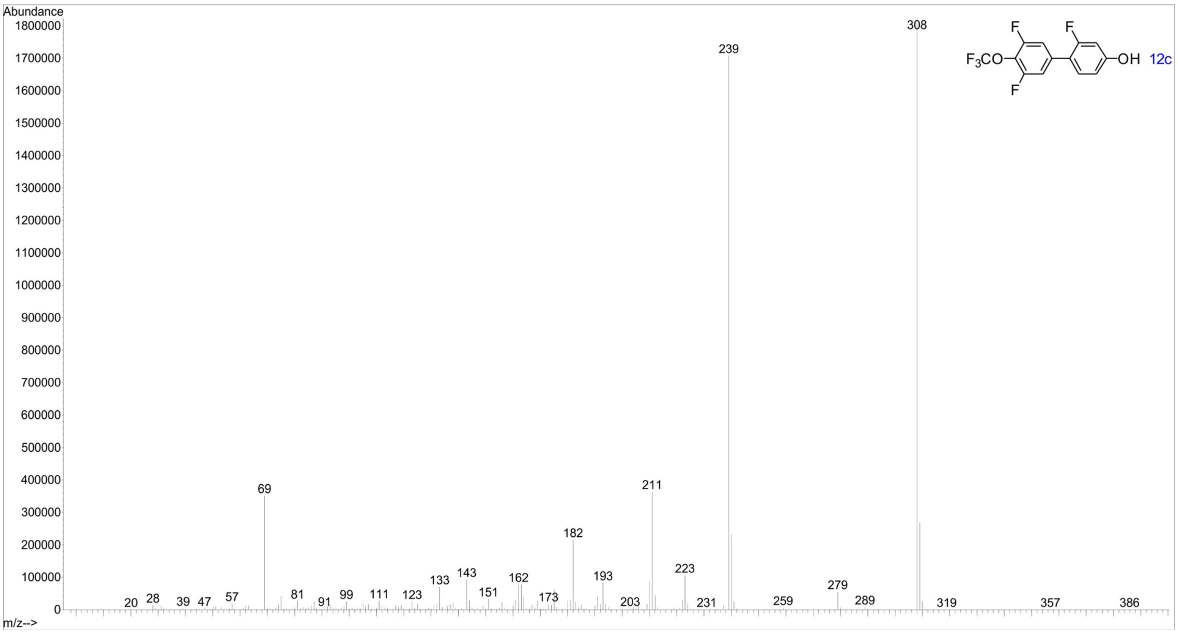

Figure S23. MS spectrum for compound of **12c**

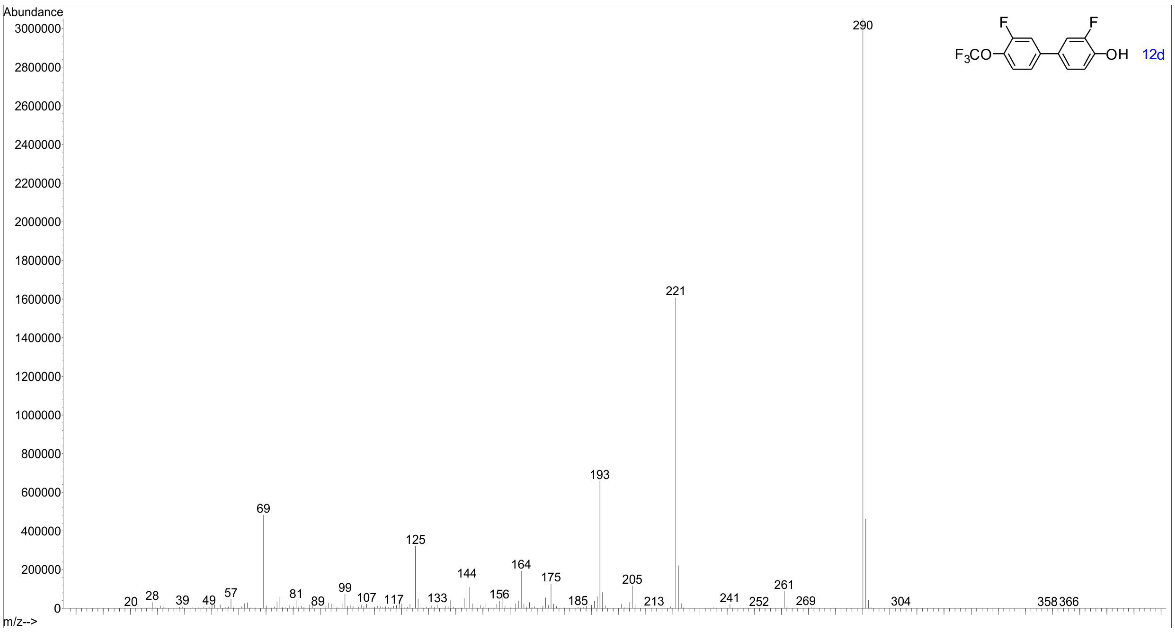

Figure S24. MS spectrum for compound of **12d**

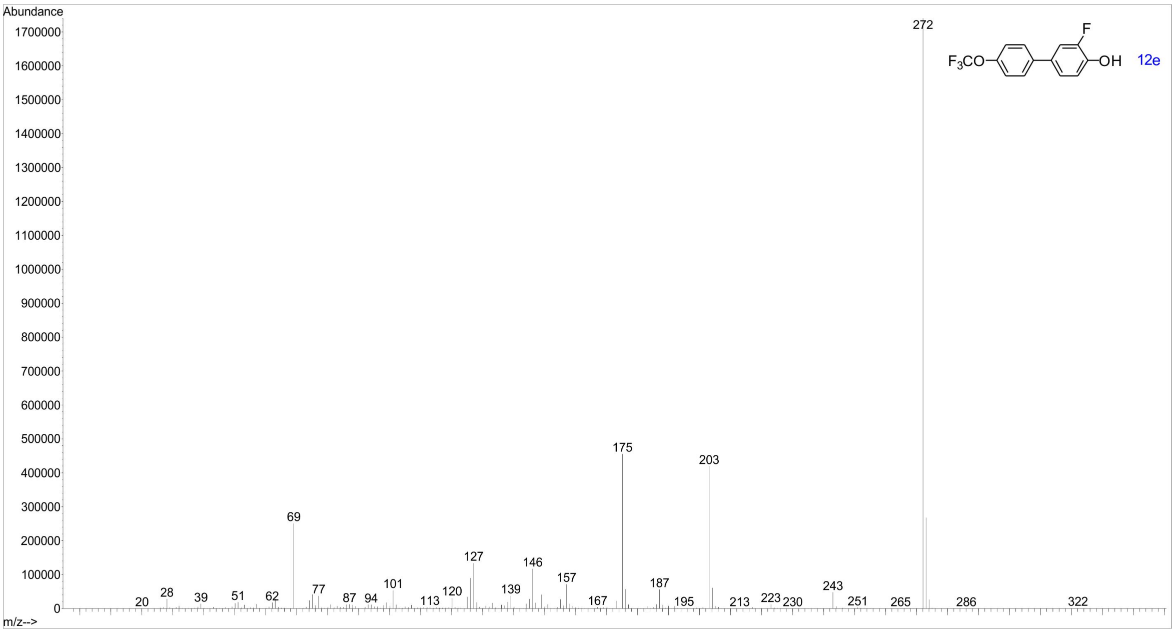

Figure S25. MS spectrum for compound of **12e**

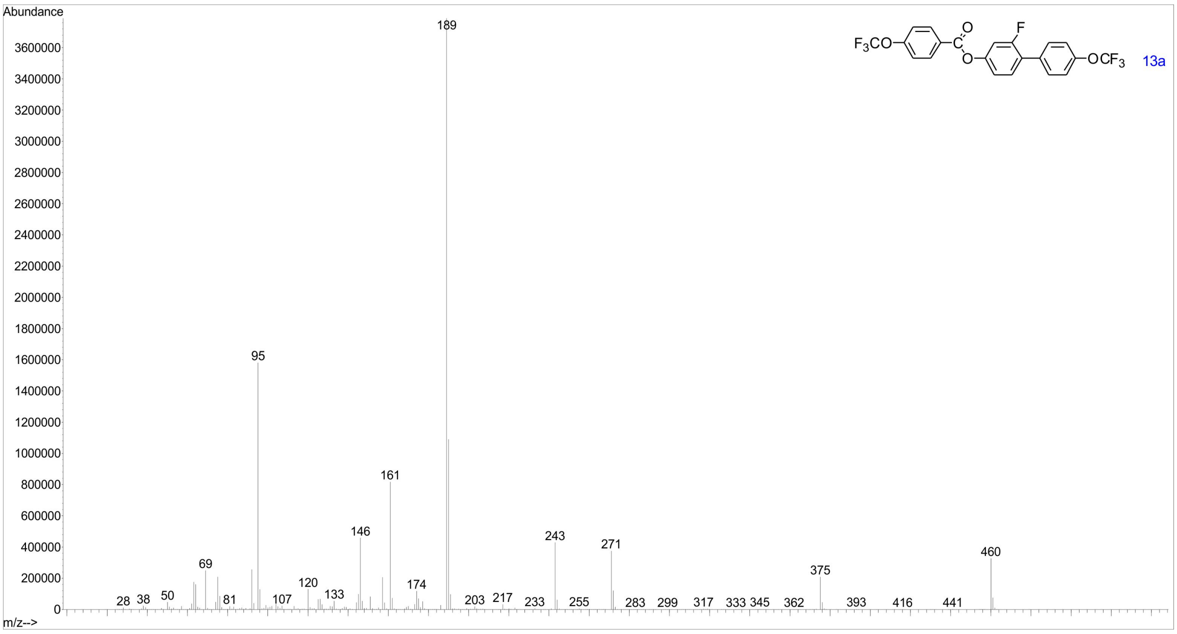

Figure S26. MS spectrum for compound of **13a**

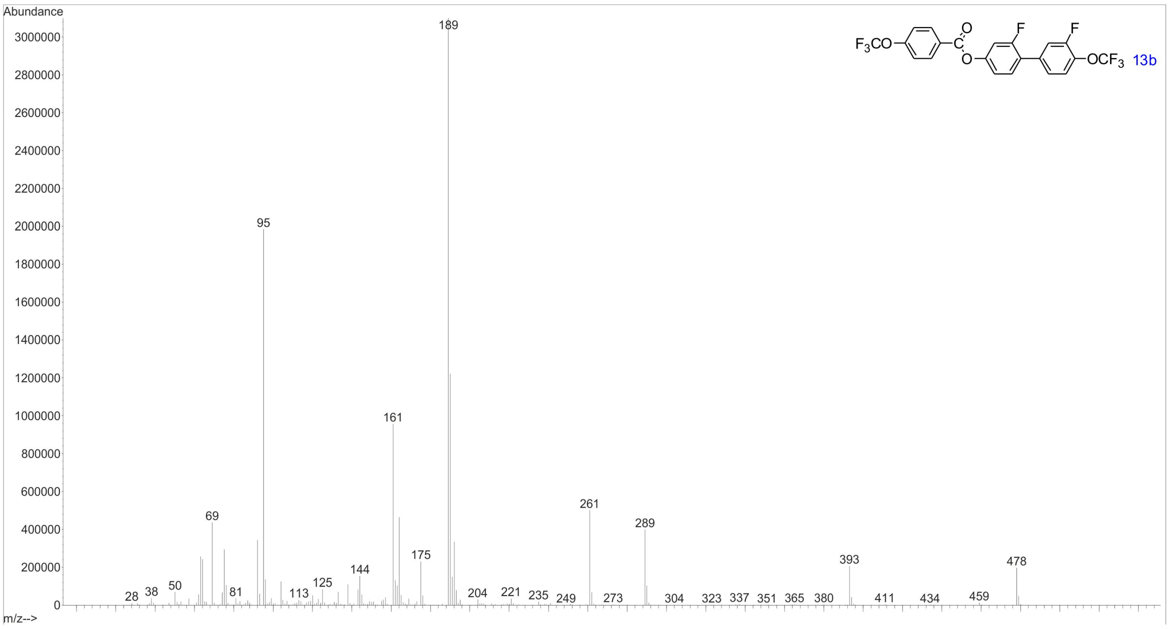

Figure S27. MS spectrum for compound of **13b**

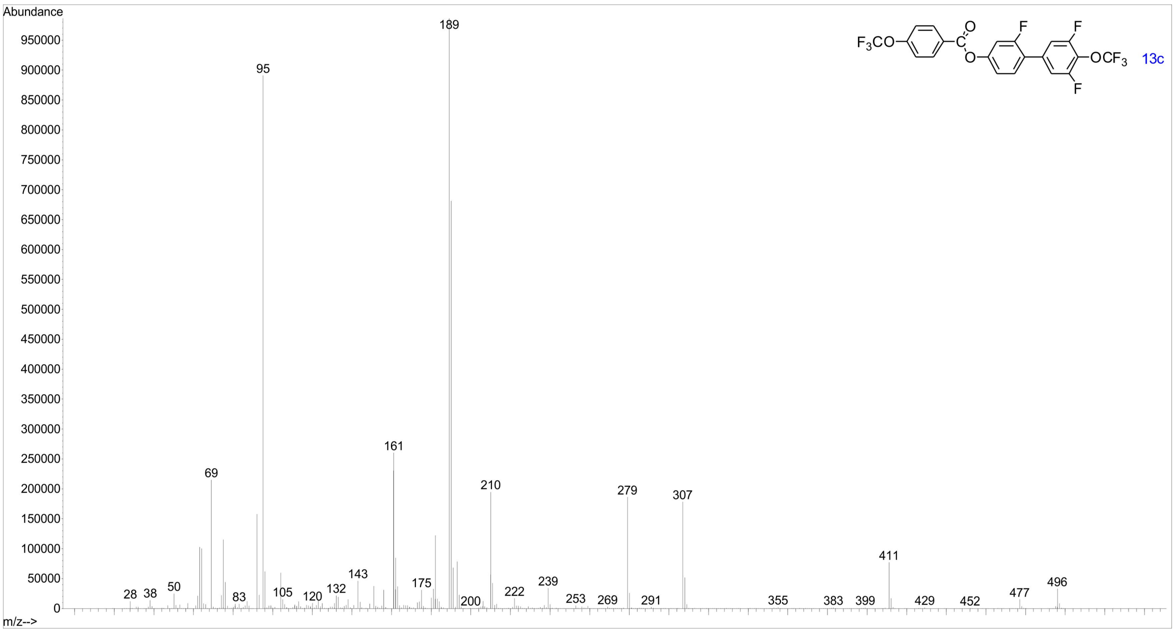

Figure S28. MS spectrum for compound of **13c**

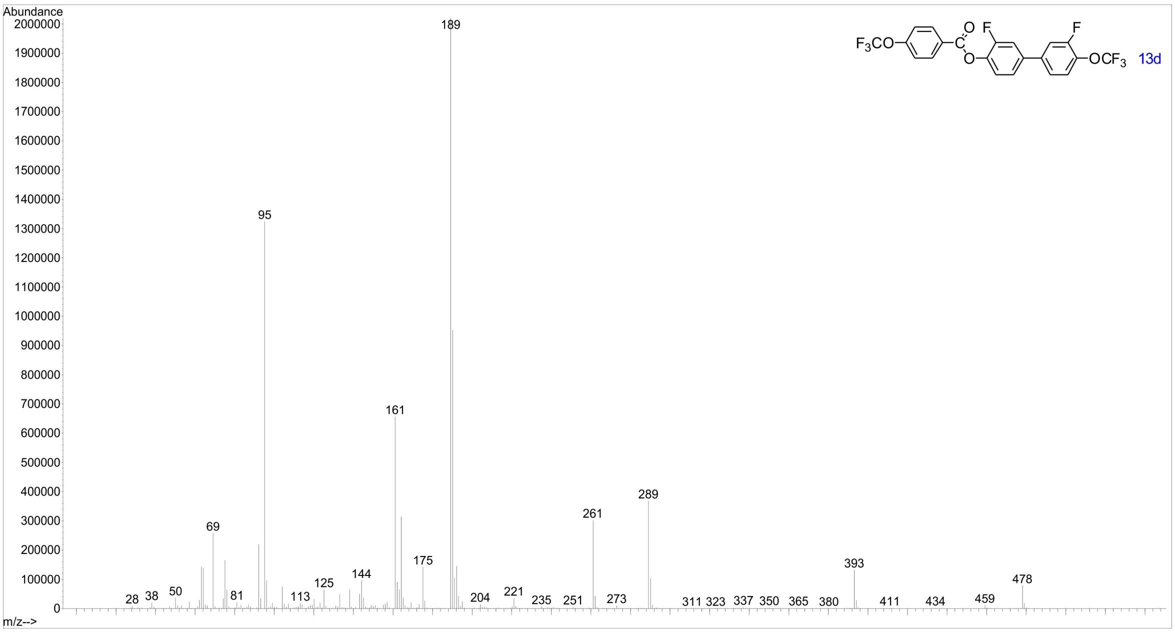

Figure S29. MS spectrum for compound of **13d**

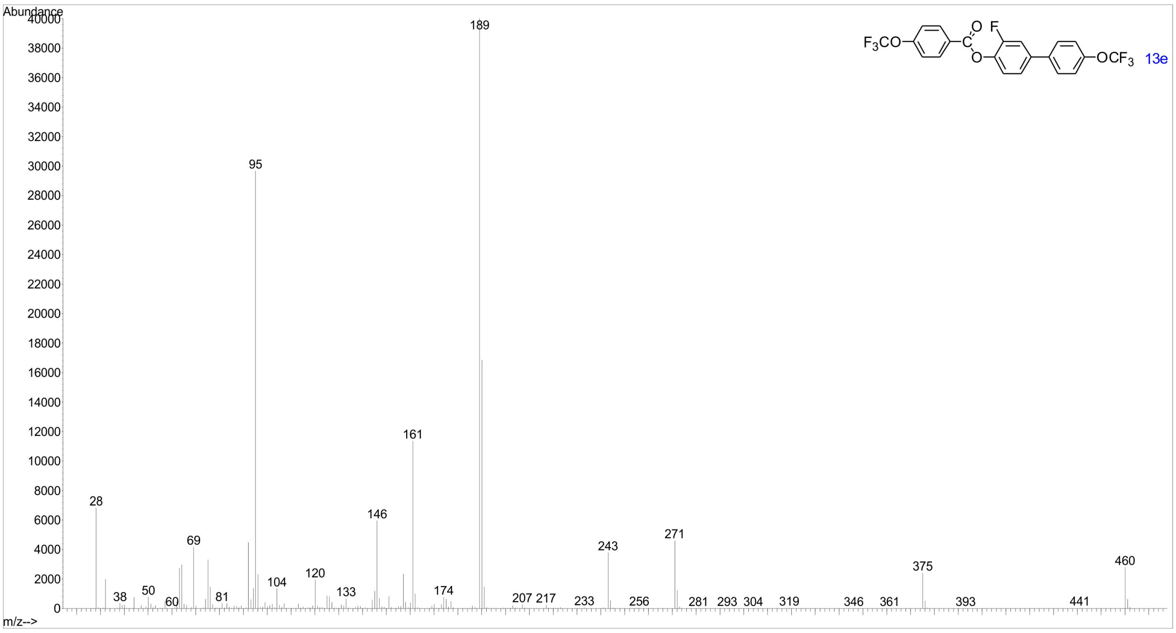

Figure S30. MS spectrum for compound of **13e**

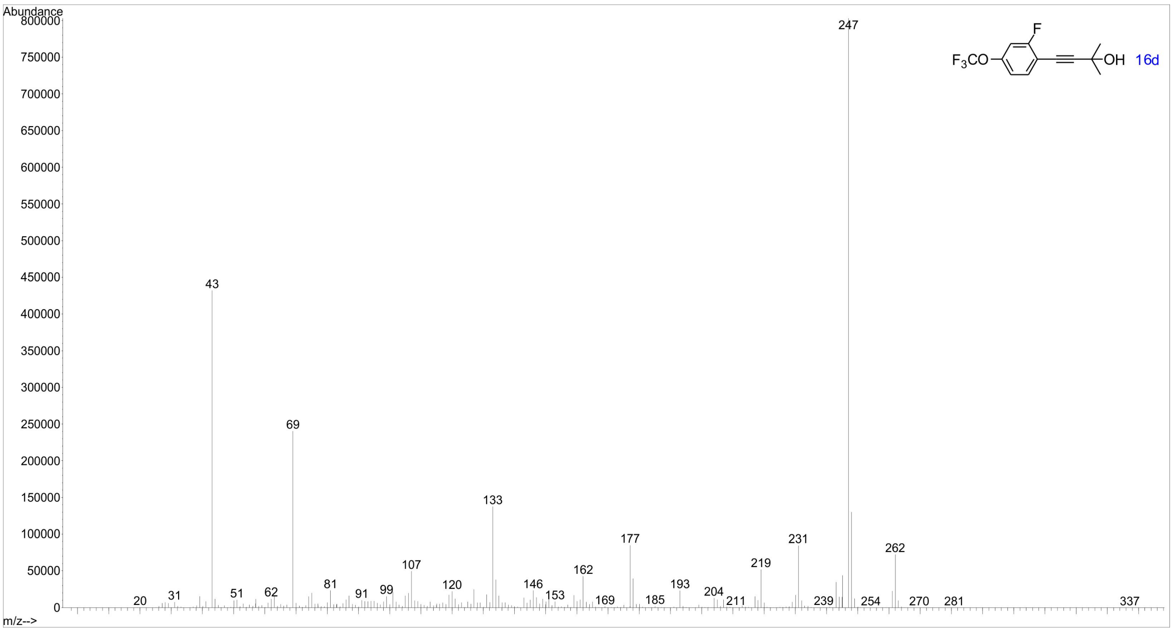

Figure S31. MS spectrum for compound of **16d**

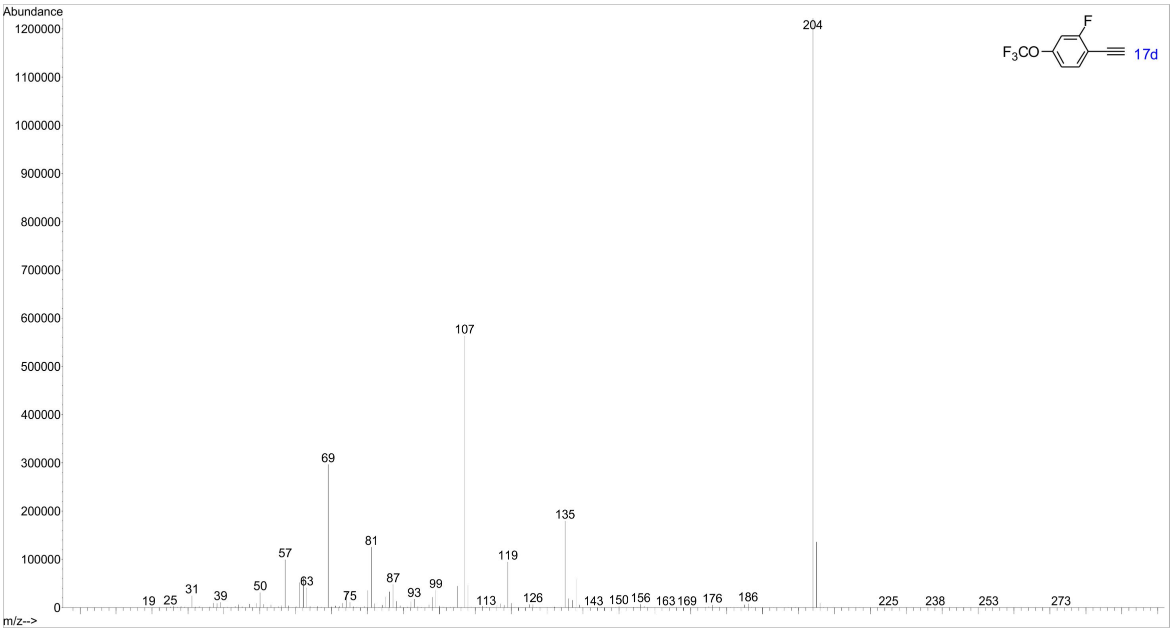

Figure S32. MS spectrum for compound of **17d**

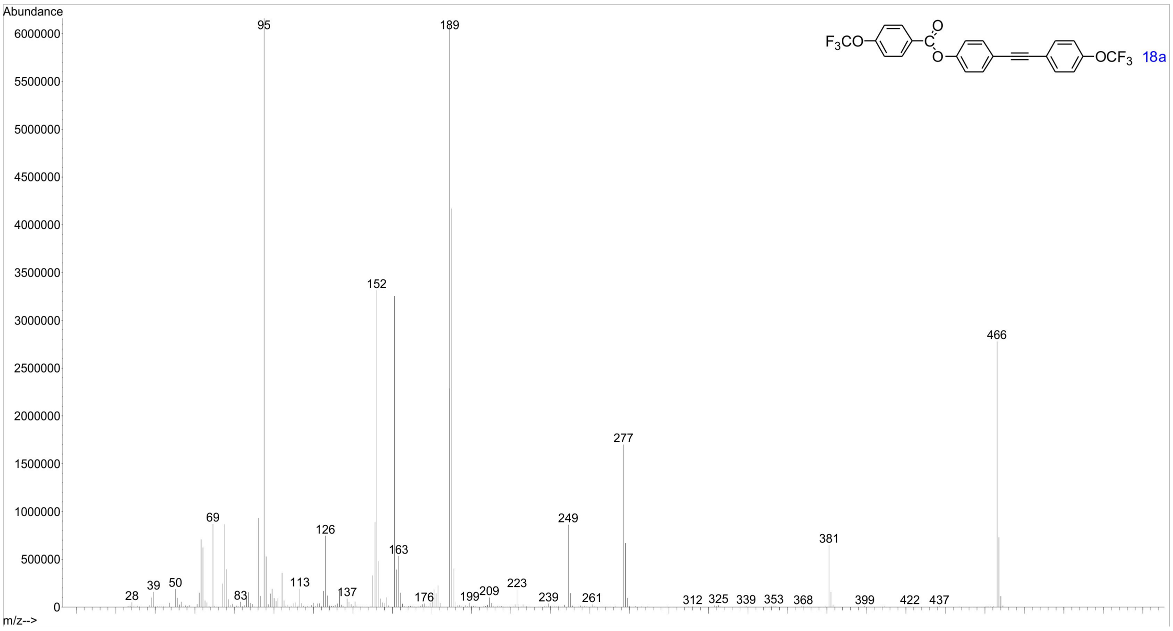

Figure S33. MS spectrum for compound of **18a**

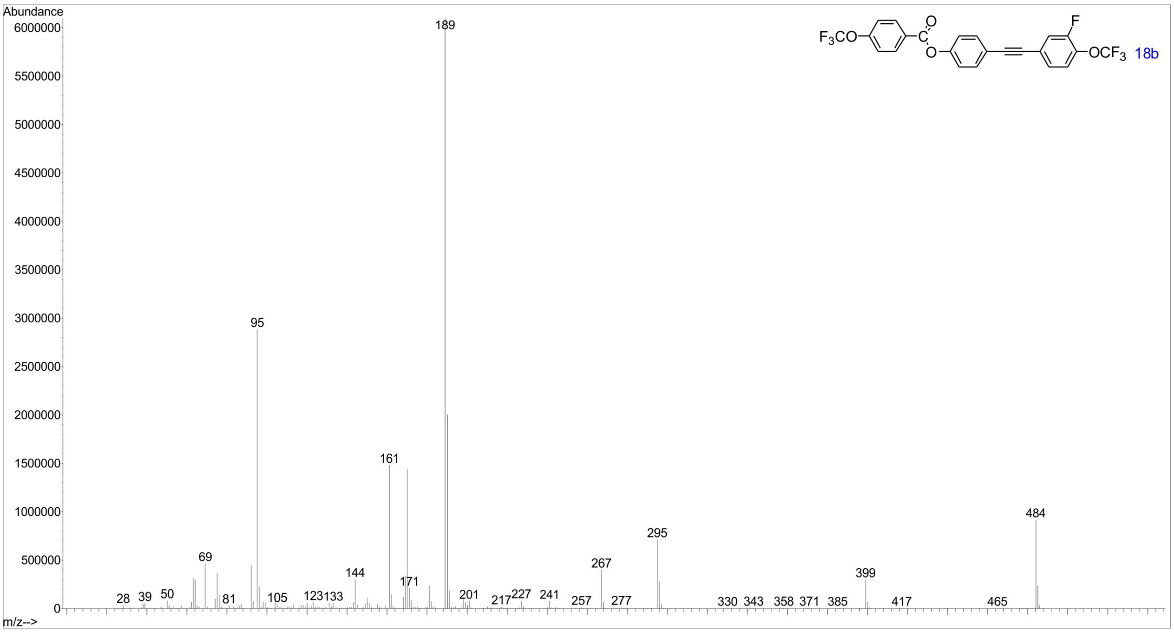

Figure S34. MS spectrum for compound of **18b**

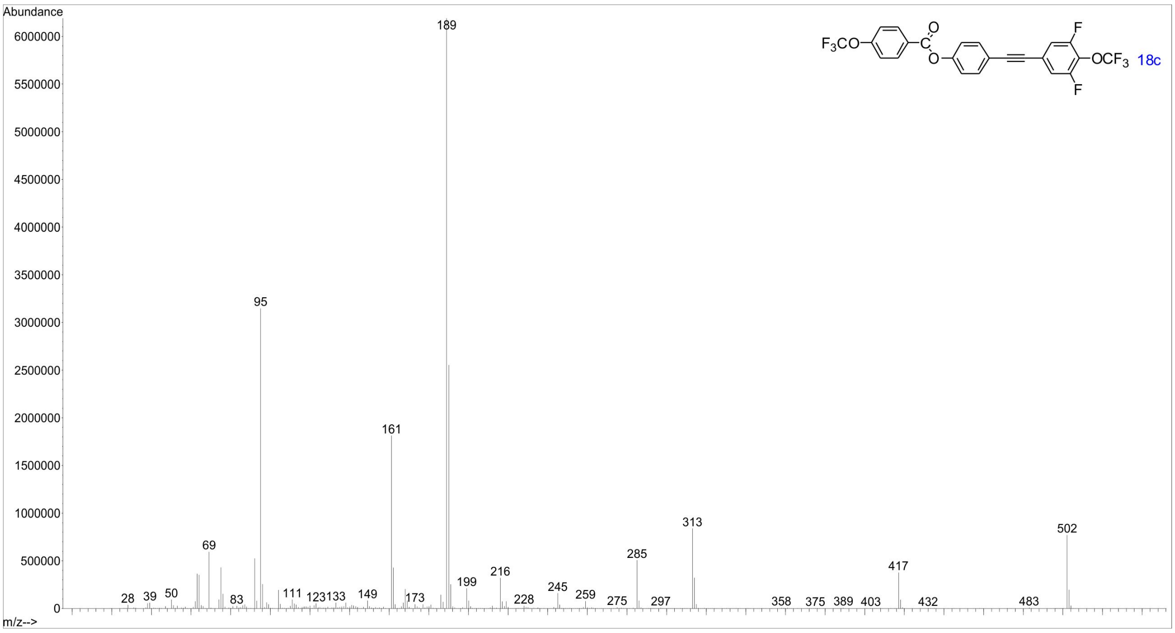

Figure S35. MS spectrum for compound of **18c**

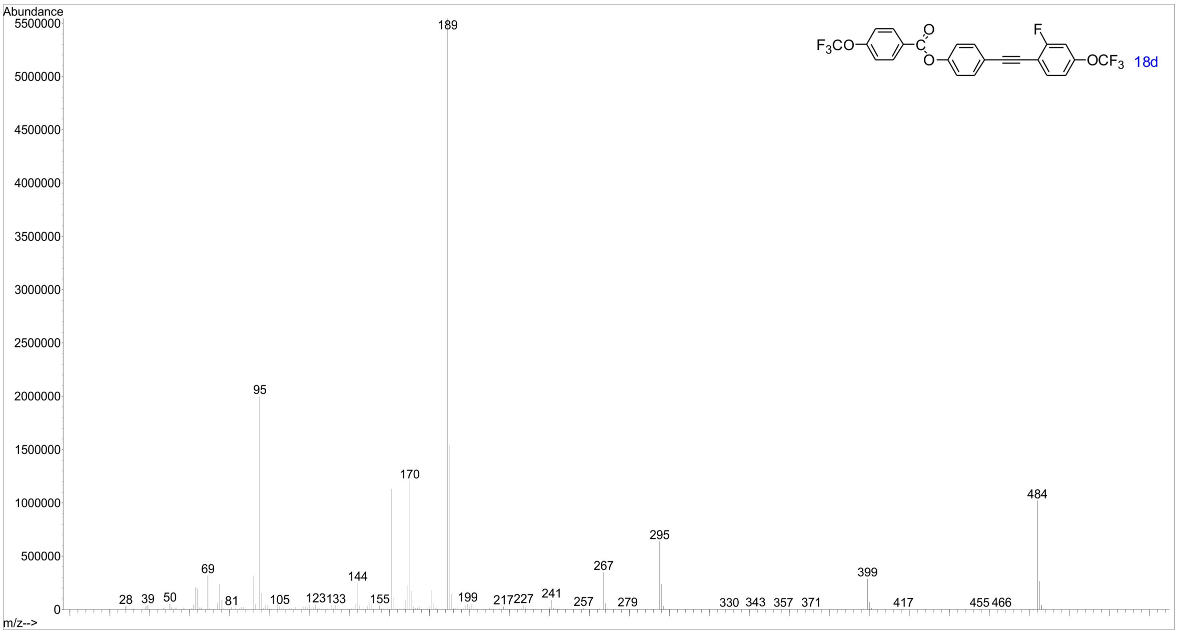

Figure S36. MS spectrum for compound of **18d**

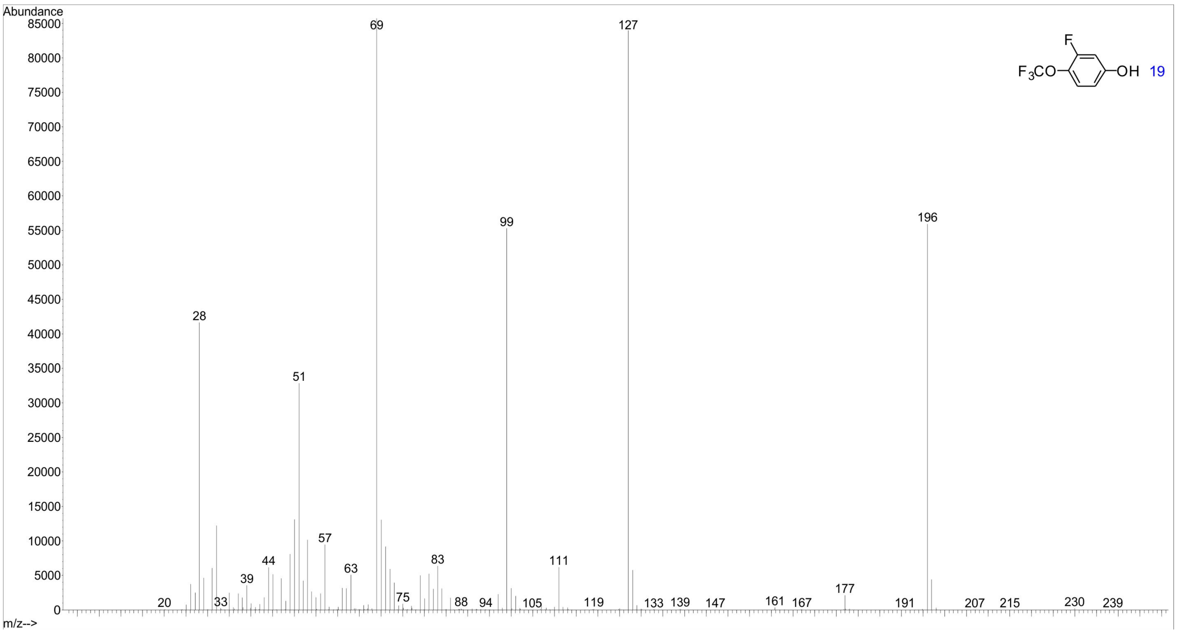

Figure S37. MS spectrum for compound of **19**

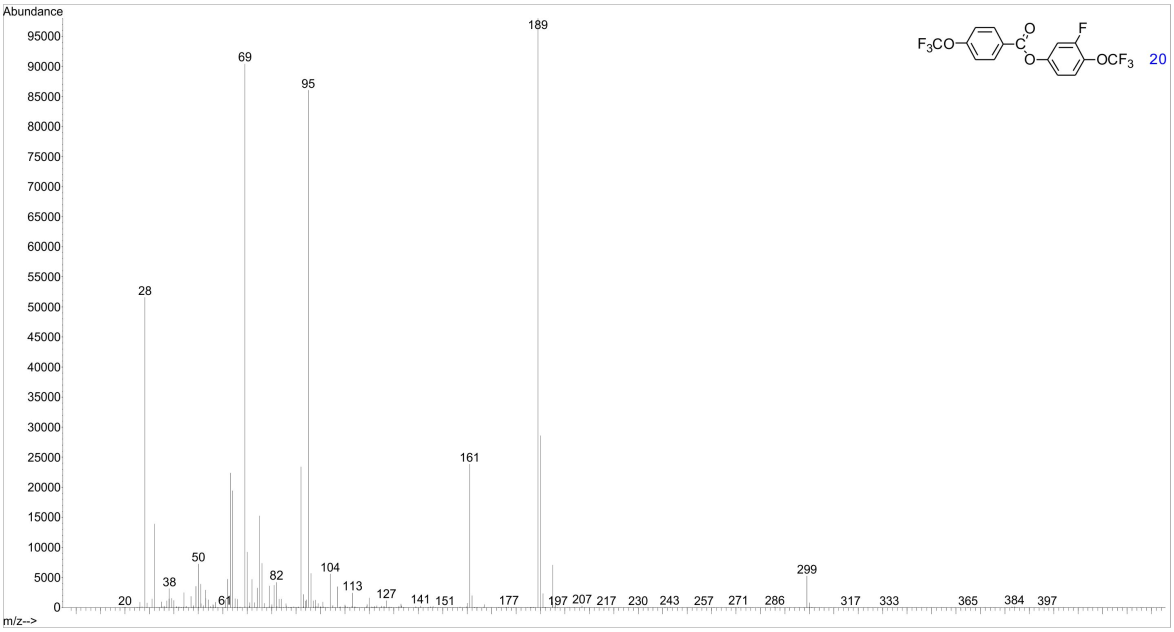

Figure S38. MS spectrum for compound of **20**

References

1. Harmata, P.; Herman, J.; Kula, P. Liquid crystals for IR: Part II synthesis and properties of perfluoroalkyl- or perfluoroalkoxy-terminated tolanes. *Liq Cryst* **2019**, doi:10.1080/02678292.2019.1606353.
